# Shedding light on *Aspergillus niger* volatile exometabolome

**DOI:** 10.1038/srep27441

**Published:** 2016-06-06

**Authors:** Carina Pedrosa Costa, Diogo Gonçalves Silva, Alisa Rudnitskaya, Adelaide Almeida, Sílvia M. Rocha

**Affiliations:** 1 Department of Biology, University of Aveiro, Aveiro, Portugal; 2 Department of Chemistry & QOPNA, University of Aveiro, Aveiro, Portugal; 3 Department of Chemistry & CESAM, University of Aveiro, Aveiro, Portugal; 4 Department of Biology & CESAM, University of Aveiro, Aveiro, Portugal

## Abstract

An in-depth exploration of the headspace content of *Aspergillus niger* cultures was performed upon different growth conditions, using a methodology based on advanced multidimensional gas chromatography. This volatile fraction comprises 428 putatively identified compounds distributed over several chemical families, being the major ones hydrocarbons, alcohols, esters, ketones and aldehydes. These metabolites may be related with different metabolic pathways, such as amino acid metabolism, biosynthesis and metabolism of fatty acids, degradation of aromatic compounds, mono and sesquiterpenoid synthesis and carotenoid cleavage. The *A. niger* molecular biomarkers pattern was established, comprising the 44 metabolites present in all studied conditions. This pattern was successfully used to distinguish *A. niger* from other fungi (*Candida albicans* and *Penicillium chrysogenum*) with 3 days of growth by using Partial Least Squares-Discriminant Analysis (PLS-DA). In addition, PLS-DA-Variable Importance in Projection was applied to highlight the metabolites playing major roles in fungi distinction; decreasing the initial dataset to only 16 metabolites. The data pre-processing time was substantially reduced, and an improvement of quality-of-fit value was achieved. This study goes a step further on *A. niger* metabolome construction and *A. niger* future detection may be proposed based on this molecular biomarkers pattern.

Filamentous fungi are eukaryotic microorganisms that are ubiquitous in nature and can affect several areas as diverse as medicine, food, and environment^1^,^2^. Some fungal species, namely, *Aspergillus* sp. can be present in hospital environments, in unfiltered air, ventilation systems, contaminated dust, water, food and ornamental plants, and can also contribute to food spoilage^3^,^4^. For instance, hospital-acquired infections are common worldwide, affecting several physiological systems, as well as surgical sites^5^. Approximately 4.1 million European patients are thought to acquire a hospital infection annually, resulting in about 37 thousand deaths^6^. Despite the low occurrence, fungal infections exhibit high rates of morbidity and mortality, mainly due to their delayed detection and treatment^7^. *Aspergillus* spp. comprises one of the most common pathogens causing fungal infections^8^, such as invasive aspergillosis^7^,^9^. Opportunistic infections by *Aspergillus niger* have increased in the last years, both in paediatric patients and adults^10^,^11^, presenting a high mortality rate, therefore strongly suggesting the need for prevention or earlier diagnosis and treatment^12^. Furthermore, *Aspergillus* species represent some concerns related to the air quality as they are the most common airborne fungi, producing allergens, which is one of the major causes of respiratory infections and can trigger allergic respiratory disorders, such as asthma, or exacerbate related symptoms^13^,^14^. *Aspergillus* species can also contaminate foods, from cultivation to harvest, during transportation and storage, increasing the likelihood of foodborne diseases, representing, therefore, a pivotal issue related to food safety^15^.

Laboratory diagnosis of fungi remains based on conventional methods, such as cell culture and subsequent identification by phenotypic, immunologic and genotypic methods^16^,^17^. However, they fail to provide as quick results as desired during life-threatening situations or economical losses due to food spoilage^18^, since the average time required for an accurate identification by culture-based methods is ranged from days to weeks^19^,^20^. Immunologic tests have been used that allow faster results, although they are not suitable for immunocompromised patients, who most frequently develop fungal infection. Molecular-based approaches also enable faster results, though they are less used in routine and applied only to specific cases^16^,^21^. Due to delayed diagnostics, physicians often initiate empirical therapies based on clinical evaluation of patients, without having specific information on etiological agent, which impairs their treatment. Thus, the development and implementation of faster, accurate and cost-effective detection tests are sorely needed^22^,^23^.

Microbial metabolomics has been breaking new ground as a useful tool in several areas, including those related to microbial detection, since microorganisms produce several volatile metabolites that can be used as unique chemical fingerprints of each species, and possibly of strains. This richness of information holds the promise for diagnosing infections *in situ* (e.g. from body fluids, food products, environmental samples, among others), circumventing the laborious recovering of microbes or their genetic material^23^,^24^. Microbial metabolomics studies have been mainly focused on the study of the volatile fraction by one-dimensional gas chromatography (1D-GC)^25^. Nevertheless, the use of comprehensive two-dimensional gas chromatography (GC × GC) has revealed that sensitivity and limits of detection are improved compared to 1D-GC^23^,^26^. Few metabolites of *A. niger* have been reported, which are distributed over numerous chemical families, such as alcohols, aldehydes, esters, ethers, ketones, hydrocarbons and terpenic compounds^27^,^28^,^29^,^30^,^31^. Despite the current instrumental developments, information of microbial metabolome is still scarce, namely that related to *A. niger*. To go further on the exploration of microbial metabolomics applications, several challenges should be overcome, since microbial culturing in representative conditions, alongside the technical difficulties to identify and/or quantify trace metabolites within complex matrixes, as well as the inherent problems related to data processing are partially responsible for the paucity of information on the full volatile metabolome of common microbial pathogens. Thus, this research aims to develop a comprehensive platform for *A. niger* detection management, contributing to the exploration of its exometabolome. An in-depth study of the headspace content of *A. niger* cultures was performed upon different growth conditions, using a methodology based on headspace-solid phase microextraction combined with GC × GC with time of flight mass spectrometry detection (HS-SPME/GC × GC-ToFMS), an advanced gas chromatographic based methodology with high resolution and high throughput potentialities. Also, Partial Least Squares-Discriminant Analysis (PLS-DA) and cross validation were performed to assess both the predictive power and classification models robustness, going a step further on exploring the potential of this methodology towards *A. niger* future detection using different fungi species and testing its reliability for genera distinction, based on the molecular biomarkers pattern achieved.

## Results and Discussion

### Evaluation of the impact of growth conditions on the *A. niger* metabolism

**Figure 1:**
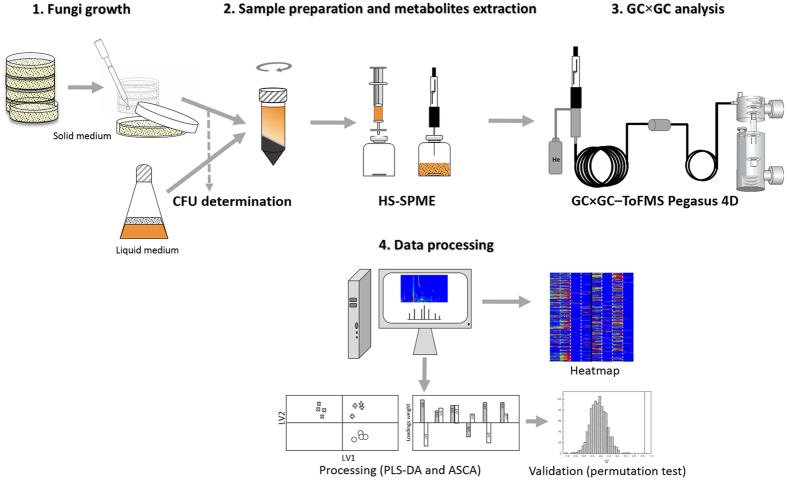
Schematic representation of the main stages for *A. niger* exometabolome determination. The stages include fungi growth, sample preparation and metabolites extraction, GC × GC analysis and data processing.

The experimental strategy was established according to the methods currently performed for fungal detection (Fig. 1), since conventional procedures leading to filamentous fungi identification are often based on fungi growth on solid media, normally for 7 days^32^,^33^. In order to reduce the identification time, in this study, two growth periods were evaluated (3 and 5 days), both comprised within the exponential phase of growth^34^. As strict aerobic fungal species, *A. niger* is commonly grown on solid media. In the present study, solid and liquid media (YGC_A_ and YGC) were used to evaluate the best approach to collect the fungal metabolites. YGC culture broth may be directly extracted using HS-SPME, but an additional extraction with Ringer solution was performed to recover the metabolites from YGC_A_. Finally, the growth temperature effect was also considered. *A. niger* is present worldwide and supports a relatively wide range of temperatures (maximum at 45–47 °C), with optimal ones at 35–37 °C, using solid media^34^,^35^. Thus, two temperatures were assayed (25 and 37 °C) since filamentous fungi are able to grow at 37 °C during a host infection and 25 °C can be used in detection of fungi contamination in food samples or in air analysis^33^.

**Figure 2:**
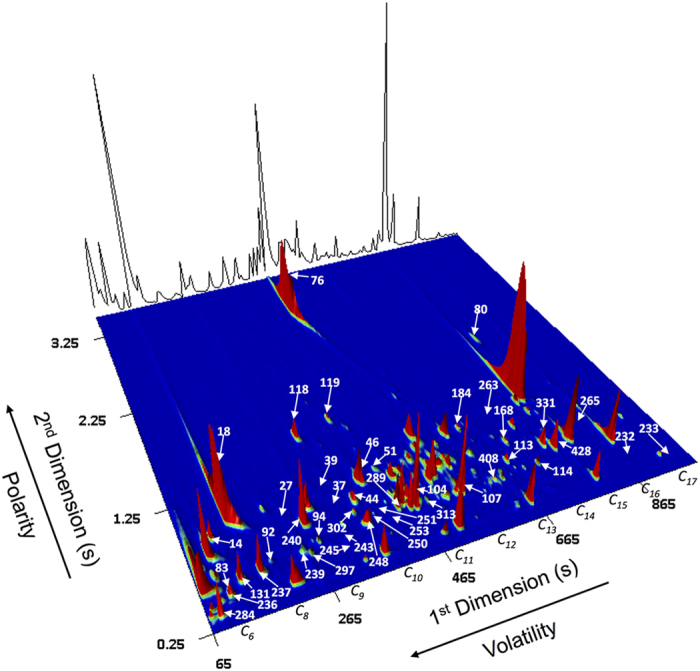
GC × GC-ToFMS total ion chromatogram contour plot of the *A. niger* culture headspace volatile components. The sub-data set of 44 metabolites defined as the *A. niger* molecular biomarkers pattern was highlighted (see peak number assignment on Table S2). Growth conditions: 3 days of growth, at 37 °C, using YGC.

After fungi growth, sample preparation and volatile metabolites extraction as well as GC × GC instrumental analysis, the data collection for matrices construction regarding further statistical analysis represents a major challenge. GC × GC peaks representing signals were selected and a peak table was constructed with GC × GC peak area data, and other useful data for analytes identification (Tables S1). The most reliable way to validate the compound identification is the co-injection of an authentic standard, though it would be unachievable in the analysis time and, in most of the cases, the standards are unaffordable or not commercially available^36^. Thus, several parameters were used as a strategy to perform a putative identification of volatile compounds based on the comparison of their mass spectra to in-house and commercial libraries (spectral similarities >800/1000) and by comparison of the RIs calculated (RI_calc_) with those reported in the literature (RI_lit_) for ^1^D column or equivalent, with differences up to 6%. Also, GC × GC structured chromatogram principle was a powerful tool in the identification procedure, since compounds structurally related should be on similar 2D chromatographic space^26^. Considering the set of columns used (Non Polar/Polar), the decreasing in volatility (high ^1^*t*_R_) is mainly related to the increasing in the number of carbons through the ^1^D. Otherwise, increasing in the ^2^*t*_R_ corresponds to polarity increasing (Fig. 2).

Total ion chromatograms were processed using the data processing software ChromaTOF®, and, on average, *ca.* 504 instrumental features were detected per sample, though only 428 were considered for dataset construction after removing the media-related compounds, such as furan-type compounds, furanones, halogenated compounds and pyrazines based on previous studies about YGC volatile composition^37^. The list of the 428 analytes was established considering all the growth conditions under study, which were distributed over the following chemical families: hydrocarbons, including aliphatic and aromatic ones (17, 5%), alcohols (17.3%), esters (15.8%), ketones (15.4%), aldehydes (10.3%), terpenic compounds (10.5%), *S*-compounds (4.2%), *N*-compounds (3.0%), ethers (2.8%), acids (2.3%) and norisoprenoids (0.7%) (Table S1, from Supplementary Information).

Few metabolites of *A. niger* have been previously identified, namely, ethanol, 1-propanol, 2-methyl-1-propanol, 2-methyl-1-butanol, 3-methyl-1-butanol, 2-pentanol, 1-octen-3-ol, 3-octanol, acetaldehyde, ethyl propanoate, ethyl butanoate, ethyl tiglate, 3-methyl-2-butenoic acid ethyl ester, iso-amyl tiglate, 3-methylbutanoic acid pentyl ester, ethyl palmitate, ethyl linoleate, 2,5-dimethoxytoluene, 2-methylfuran, 3-methylfuran, 2-propanone, 2-pentanone, 2-heptanone, 3-octanone, cyclohexanone, heptane, 2,3,3,3-tetramethylbutane, 1,3-nonadiene, pentadecene, limonene, α-bisabolene and α-cubebene^27^,^28^,^29^,^30^,^31^. These compounds have been detected using different sampling/extraction tools, such as Tenax® adsorbent trap^27^, SPME with non-polar or bipolar stationary phases^28^,^29^,^30^ and passive sampling onto charcoal sorbents^31^, followed by thermal desorption into 1D-GC. As far as we know, the present study represents the first one using GC × GC, and its high sensitivity and chromatographic resolution revealed the high complexity of the *A. niger* cultures headspace (Table S1).

**Figure 3:**
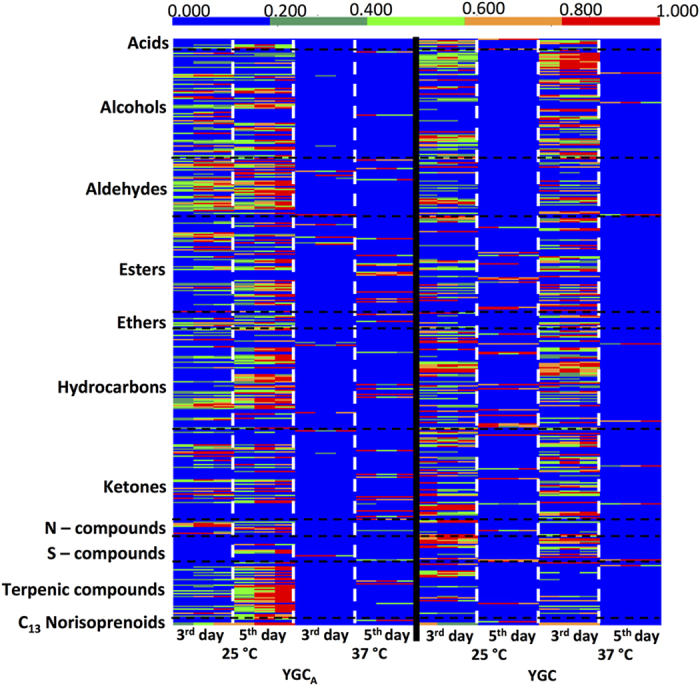
Heatmap representation of the 428 metabolites putatively identified from *A. niger* cultures. The heatmap comprises the data obtained from the cultures, upon different growth conditions, such as culture medium (solid and liquid YGC), temperature (25 and 37 °C) and incubation period (3 and 5 days). Each variable area was normalized by the maximum of each metabolite for all samples (with GC peak previously normalized by CFU mL^−1^). *n* = 3 for each condition under study.

For an easy, quick and global assessment of the impact of the growth conditions on the *A. niger* metabolism, a heatmap representation (Fig. 3) was prepared, in which GC peak areas (previously normalized by CFU mL^−1^) were normalized by the maximum of each metabolite. Each compound was illustrated through different colour intensities, allowing a visual assessment of the relative abundance of each chemical family from the putatively identified compounds. As cell growth was dependent on the conditions under study, in order to compare the volatiles pattern for all conditions under study and expressed their content as area/cell concentration, their content was normalized by CFU mL^−1^. For YGC_A_, higher cell concentration (expressed by CFU mL^−1^) was observed at 37 °C compared to 25 °C (Fig. S1, from Supplementary Information), as previously reported^34^,^35^. For YGC, the growth period (3 and 5 days) seems to have higher impact on cell concentration rather than temperature.

Heatmap revealed the high complexity of *A. niger* cultures headspace, mainly composed by their exometabolome components, and their distinctive relative abundance may be explained by different constraint affecting the metabolic pathways of *A. niger*, upon different conditions, namely temperature and growth time. The major relative content of total metabolites per CFU mL^−1^ was observed for 5 days of growth, at 25 °C, using YGC_A_. Within this condition, it was also observed the major relative abundance of terpenic compounds. Higher relative abundances of alcohols, aldehydes, hydrocarbons and ketones were observed at 25 °C, for both incubation periods using YGC_A_, whereas higher relative abundances for liquid experiments were seen at 3 days of growth, for both temperatures under study.

To assess the influence of the studied conditions over metabolite production, ASCA was applied to the dataset comprising 428 variables. According to the results, all the main effects and their interactions were statistically significant (*p* < 0.05). The culture medium (*p* < 0.0005) was the condition that exhibited the higher impact, explaining 26.7% of the total dataset variance; followed by the temperature (*p* = 0.0045) and growth period (*p* = 0.006) conditions, explaining 12.7% and 12.8% of the variance, respectively. The interaction effects culture medium × temperature and culture medium × growth period were statistically significant (*p* = 0.0035) as well, each explaining 5.7% of the overall variance. The interaction temperature × time was also significant (*p* = 0.024) explaining 4.1% of the variance. The significance attributed to media used by ASCA analysis is in accordance with previous studies. In the case of solid medium use, the physical proximity of the colonies increase from three to five days of incubation (which do not always occur in liquid medium), affecting the colony size and consequently the *A. niger* metabolism. The morphology of the colonies impacts the production of enzymes and metabolites^35^. However, the mechanisms underlying the impact of morphology on productivity is not yet clear^38^. At the same time, the center of large colonies may experience oxygen starvation and other nutrients may also become limiting in this part of the mycelium. These gradients are expected to be less pronounced during dispersed growth in liquid medium^39^. Moreover, surface sensing due to colony size and physical proximity can also regulate the fungi metabolism, increasing signalling between hyphae. It has been observed that the heterogeneity of expression of genes at the periphery of the macro-colonies is more robust than that in micro-colonies cultivated in liquid medium. In contrast to solid media, gradients of signalling molecules cannot be formed between hyphae that are grown in liquid cultures^38^. Despite providing the appropriate nutrients into the culture medium, it is also necessary to attend the biophysical requirements during fungal growth, such as oxygen levels, temperature and also the growth time. As the growth of *A. niger* only occurs under aerobic conditions, since all filamentous fungi are strict aerobes, its growth on YGC_A_ was allowed to occur throughout a bigger surface area than that of Erlenmeyers containing YGC (Fig. S1), thus solid medium condition promotes biggest surface areas and enables higher oxygen exposure to the surface, allowing fungi to grow under more convenient environment. On the other hand, toxic compounds resulting from the aerobic respiration can be accumulated in the culture medium affecting the fungi growth, namely during long periods of incubation and when the fungi are in contact with these toxic compounds, such as observed after 5 days of incubation on solid medium. Moreover, even with an additional extraction step by recovering the contents of YGC_A_ with Ringer solution, where losses or incomplete metabolites recovery were thought to occur, the results show that a larger number of compounds were detected in Ringer extract when compared to YGC experiments, where the volatiles profiling was performed using the whole cell-free broth.

### Establishment of *A. niger* molecular biomarkers pattern

A sub-data set of 44 metabolites putatively identified in all studied conditions was defined as *A. niger* molecular biomarkers pattern (Table 1), belonging to hydrocarbons, including aliphatic and aromatic ones (31.8%), alcohols (22.7%), aldehydes (20.5%), ketones (11.4%), esters (6.8%), terpenic compounds (4.6%) and norisoprenoids (2.3%). Also, in order to confirm the origin of these analytes, their content in the *A. niger* cultures and medium control for the same condition (YGC and YGC_A_ rinsed with Ringer solution, in all the tested conditions: 25 °C and 37 °C, both for 3 and 5 days) were compared. From the sub-set of 44 analytes, 4-bis(1,1-dimethylethyl)-phenol; hexadecane; heptadecane; 2-methyl-6-phenyl-1,6-heptadiene; 3-nonen-2-one; 2,6-dimethyl-7-octen-2-ol were not detected in the media for all the conditions under study. The others 38 analytes were detected in the media in levels at least 3 standard deviations lower than in the *A. niger* culture for similar condition, confirming that their origin may be associated with fungus metabolism.

The *A. niger* molecular biomarkers pattern comprises some metabolites already reported for this species, such as 1-hexanol, 1-heptanol, 1-octanol, 3-methyl-1-butanol and hexanal^40^. Compounds like 1-octen-3-ol and 3-octanol were also achieved within this pattern, which have already been reported as fungi-related compounds^28^.

**Figure 4:**
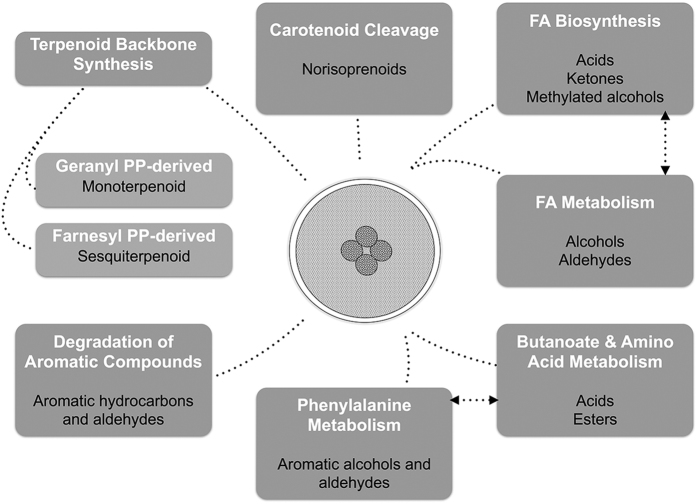
Schematic representation proposed to explain *A. niger*metabolic pathways related to molecular biomarkers pattern chemical families^25^,^36^,^37^,^38^,^39^. FA – Fatty Acid; PP – Diphosphate.

This sub-set of metabolites may arise from several pathways, and a schematic representation was proposed in order to arrange this information (Fig. 4), which reveals that a network of pathways is involved to explain the *A. niger* molecular biomarkers pattern. Some of the metabolites are known to play important roles in degradation of aromatic compounds pathway, such as toluene, 1,2-dimethylbenzene, benzaldehyde, benzylalcohol, ethylbenzene and acetophenone. Also, acids like butanoic acid, 2-methylbutanoic and 3-methylbutanoic acids are connected to butanoate and amino acid metabolism. Acetaldehyde, 2-phenylethanol and benzeneacetaldehyde are related to phenylalanine metabolism. Other chemical families are associated with biosynthesis of unsaturated fatty acids, such as acids, ketones and methylated alcohols. Aldehydes and alcohols have also been related to fatty acids metabolism^27^,^40^,^41^. α-Pinene, 1,8-cineole, linalool, limonene, menthol and α-terpineol belong to a group of diverse chemical compounds, consisting of two isoprene units derived from geranyl diphosphate, which are related to the monoterpenoid biosynthesis pathway. Pinocarveol and pinocarvone, also detected in this study, are derived from the metabolism of α-pinene. On the other hand, sesquiterpenic compounds consist of three isoprene units derived from farnesyl diphosphate, which are also related to terpenoid backbone synthesis, such as longifolene (humulene type), α-bisabolene (bisabolene type) and the acyclic nerolidol. The C_13_-molecules norisoprenoids can be derived from the cleavage of carotenoids^42^,^43^.

### Going further on genera distinction

An exploratory study was done to evaluate the potential of the established molecular biomarkers pattern of *A. niger* (Table 1) though from different genera. *Penicillium chrysogenum* was chosen to compare two filamentous fungi, though from different species, and *Candida albicans* was selected for its importance among immunocompromised patients within clinical setting^8^.

The sub-data set of 44 metabolites was present in all the three fungi under study, though differences were registered: 1-heptanol was not present in *P. chrysogenum* for 3 and 5 days of growth, whereas 1-butanol and 3-nonen-2-one were not present in *C. albicans* isolates for 5 days of growth.

**Figure 5:**
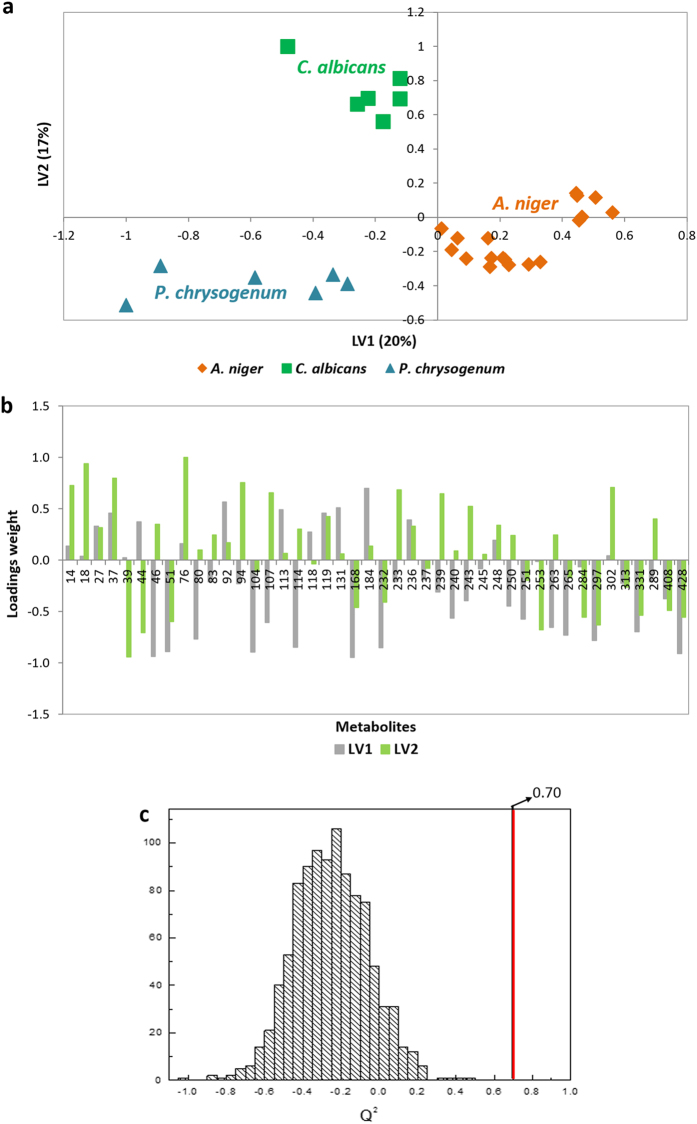
Statistical analysis applied to the sub-data set comprising 44 metabolites. (**a**) PLS-DA scatter score plot (LV1 × LV2) and respective (**b**) loading plot of *A. niger* (orange rhombs), *C. albicans* (green squares) and *P. chrysogenum* (blue triangles), using a sub-data set comprising 44 metabolites putatively identified in all studied conditions by HS-SPME/GC × GC-ToFMS (peak identification is presented in Table S2 from Supplementary Information), with GC peak areas normalized by CFU mL^−1^ and variables numbered accordingly. (**c**) Q^2^ values distribution for the permuted model (1000 permutations) and for the initial model. Permutation test was applied, and a statistically significant model for 44 metabolites was obtained with a predictive Q^2^ capability of 0.70 for *A. niger. n* = 3 for each condition under study.

PLS-DA scores scatter plots were used for visualizing the variability between samples, and the corresponding loadings to access the variables that explain the distinction. Thus, Fig. 5a,b represents the PLS-DA LV1 × LV2 scores and loadings scatter plots, respectively. LV1 and LV2 explain *ca*. 37% of the dataset variability (Fig. 5a) and the separation of the filamentous fungi is obtained by LV1 axis, where *P. chrysogenum* and *A. niger* are dispersed throughout the LV1 negative and LV1 positive, respectively; whereas *C. albicans* is dispersed throughout LV2 positive. Thus, discrimination between the different microorganisms under study was clearly observed. Different metabolites contributed to the fungi distinction, as stated on the loadings plot (Fig. 5b). Statistical significance of PLS-DA classification model was assessed using permutation test and Q^2^ as a quality-of-fit criterion (Fig. 5c). Distribution of the Q^2^ values for *A. niger* class membership prediction for initial model and 1000 permuted models and the Q^2^ of the initial model are shown on Fig. 5c. Q^2^ values for initial models were 0.70 for *A. niger*, 0.68 for *C. albicans* and 0.80 for *P. chrysogenum*. Q^2^ values for all permuted models were below the ones of the initial models, allowing one to conclude that the initial model was not random (*p* < 0.001).

The metabolites playing important roles in the above-mentioned classification model were chosen according to the VIP values (Variable Importance in Projection). Thus, another sub-data set was constructed comprising 16 metabolites with VIP values higher than 0.8 (Table 2) and another PLS-DA was performed. VIP analysis allowed to decrease the number of variables in the model and to improve model performance as well (Q^2^ value of 0.8). Thus, the pre-processing (to go from raw instrumental data to clean data for data processing) time was substantially reduced, as it was only extracted the raw instrumental data from 16 metabolites.

**Figure 6:**
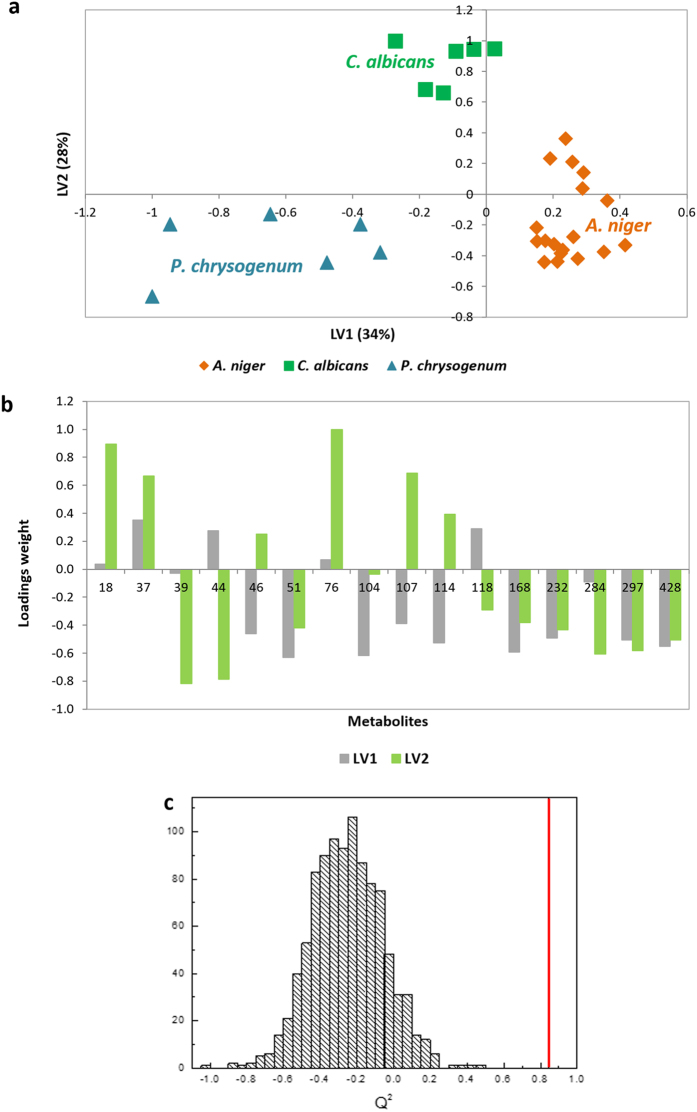
Statistical analysis applied to the sub-data set comprising 16 metabolites with VIP value higher than 0.8. (**a**) PLS-DA-VIP score scatter plot (LV1 × LV2) and respective (**b**) loading plot of *A. niger* (orange rhombs), *C. albicans* (green squares) and *P. chrysogenum* (blue triangles), using a sub-data set comprising 16 metabolites (with VIP value higher than 0.8) putatively identified in all studied conditions by HS-SPME/GC × GC-ToFMS (peak identification is presented in Table S1), also with GC peak areas normalized by CFU mL^−1^ and variables numbered accordingly. (**c**) Q^2^ values distribution for the permuted model (1000 permutations) and for the initial model. Permutation test was applied, and a statistically significant model for 16 metabolites was obtained with a predictive Q^2^ capability of 0.86 for *A. niger. n* = 3 for each condition under study.

PLS-DA model calculated with 16 metabolites comprised 4 latent variables instead of 5 as the model calculated with 44 metabolites. The PLS-DA scatter plot (Fig. 6a) shows a clear distinction between fungi under study, through the contribution of different compounds (Fig. 6b). Optimized classification model also possessed improved prediction capability with Q^2^ values of 0.86 for *A. niger*, 0.86 for *C. albicans* and 0.85 for *P. chrysogenum*. Classification model was found to be statistically significant according to the permutation test (Fig. 6c).

In summary, the methodology based on HS-SPME/GC × GC-ToFMS tandem with PLS-DA and respective cross validation provides novel data on *A. niger* exometabolome. Despite the advantages of the advanced multidimensional gas chromatography, HS-SPME using divinylbenzene/carboxen™/polydimethylsiloxane StableFlex™ (DVB/CX/PDMS) is also a crucial step for metabolites profiling. DVB/CX/PDMS is an adsorbent fiber combining porous polymer, a carbon molecular sieve, and PDMS, being recommended for non-targeted analysis. It is a bipolar fiber with high extraction efficiency for a wide range of volatile molecules, namely hydrophilic and hydrophobic ones. SPME is a non-exhaustive extraction technique, thus it is recommended that SPME experimental parameters should be carefully controlled to ensure that all samples are analysed under the same conditions and consequently, respective data may be compared. The use of other sorbent materials and tools may not be excluded in further studies. For instance, Purge and Trap is a fast and efficient tool for volatiles extraction, presenting high sensitivity and reproducibility, however exhibits some drawbacks, i. e., a particular device is needed for sample GC introduction, not currently available in labs. Also, especial attention should be done to avoid potential sources of errors, namely, sample storage, trap-heating effects, and carryover and purging efficiency^44^.

The *A. niger* molecular biomarkers pattern was established, comprising the 44 metabolites present in all studied conditions, which may arise from several metabolic pathways of *A. niger,* such as butanoate and amino acid metabolism (acids and esters), degradation of aromatic compounds (aromatic hydrocarbons and aldehydes), biosynthesis of unsatured fatty acids (acids, ketones and methylated alcohols) and to their metabolism (aldehydes and alcohols), mono and sesquiterpenoid biosynthesis and carotenoid cleavage. This pattern was successfully used to distinguish *A. niger* from other fungi (*Candida albicans* and *Penicillium chrysogenum*) with 3 days of growth by using Partial Least Squares-Discriminant Analysis (PLS-DA), which permitted to provide a statistically significant model with predictive Q^2^ capability of 0.70 for *A. niger.* In addition, PLS-DA-Variable Importance in Projection was applied to highlight the metabolites playing major roles in fungi distinction; decreasing the initial dataset to only 16 metabolites, substantially reducing the data pre-processing time and improving the quality-of-fit value (predictive Q^2^ capability of 0.86 for *A. niger*).

This methodology can be further exploited and applied in *A. niger* detection based on a molecular biomarkers pattern. Hence, based on the developed metabolomic workflow described herein, further research in a broader set of *A. niger* collected from different conditions (clinical and environmental samples, food products, among others), might be valuable to study the *A. niger* biodiversity and to contribute for microbial platform construction, useful for a more global fungal management. As far as we know, this study represents the most detailed study about *A. niger* exometabolome, representing, therefore, an improvement towards the construction of a *A. niger* omics pipeline, that will comprise data from the combination of different type of samples and techniques (namely, NMR and LC-MS) to gain a broad perspective of fungal metabolome. Finally, it is important to point out that, microbial metabolomics represents an avenue for microorganism’s insight and this research work represents a relevant contribution. Regarding its application in particular scenarios, it is mandatory to look for other parameters, such as, reduction of the growth time and limits of detection, evaluation of confounders, matrix effects (using real matrices), and co-cultures, among others.

## Materials and Methods

The sampling, reporting of chemical analysis and metadata relative to data pre-processing, pre-treatment, processing, validation and interpretation were performed according to the Metabolomics Standards Initiative (MSI)^45^,^46^,^47^. The main stages for *A. niger* exometabolome determination are visually provided (Fig. 1), including fungi growth, sample preparation and metabolites extraction, GC × GC analysis and data processing, which are described in detail in the following sub-sections.

### Fungal species and growth conditions

Three fungi were used in this study: *Aspergillus niger* (GenBank accession number KT964850), *Penicillium chrysogenum* (GenBank accession number KT799549) and *Candida albicans (*GenBank accession number SC5314). Fresh cultures were obtained by streaking each species on Yeast Glucose Chloramphenicol Agar (YGC_A_ −20 gL^−1^ D-glucose, 5 gL^−1^ yeast extract, 0.1 gL^−1^ chloramphenicol and 18 gL^−1^ agar; Liofilchem^®^, Italy).

Firstly, to evaluate the impact of growth conditions on metabolite production, *A. niger* was incubated in different media (solid and liquid YGC), temperatures (25 and 37 °C), as well as incubation periods (3 and 5 days). Using solid YGC_A_, 5 plates were performed for each assay, and for liquid YGC experiments, 1 flask was prepared suspending the cultures in 100 mL of YGC (10 gL^−1^ D-glucose and 5 gL^−1^ yeast extract). Three independents assays were done for each condition, corresponding to a total of 15 plates and 3 flasks per condition.

For each assay, considering YGC_A_ experiments, the sampling was performed by adding 10 mL of Ringer solution (Merck Millipore) per plate (5 plates per assay) to remove the cellular content of each sample. Therefore, *ca*. 50 mL were collected from each assay, an aliquot of 25 mL was collected to volatile components profiling and other aliquot of 25 mL for cell concentration determination. For samples incubated in YGC, from each flask, the fungal sampling was performed by collecting 25 mL for volatile components profiling and 25 mL for cell concentration determination. The cell concentration, expressed as colony-forming units per millilitre (CFU mL^−1^), was determined after samples homogenization by mashing. The homogenized suspension was serially diluted in Ringer solution and aliquots of 100 μL were spread on YGC_A_ (5 replicates per dilution). These results were used to normalize the total areas of each chemical feature detected, therefore allowing the determination of specific metabolite production per cell.

Finally, to assess genera distinction based on metabolomic data, *P. chrysogenum* and *C. albicans* were also plated onto YGC_A_ at 25 °C, using two incubation periods (3 and 5 days), also performing 5 plates for each assay under study (in a total of 15 plates per condition corresponding to 3 independents assays) and the same procedure which for *A. niger* samples in solid media mentioned above was applied.

### Profiling of *A. niger* cultures headspace components

After incubation, 25 mL of each sample (YGC culture broth and Ringer extract obtained from YGC_A_, and respective medium control) were collected and centrifuged at 10.000 rpm, at 4 °C for 15 min (Centrifuge Beckman AVANTI). For HS-SPME procedure, 20 mL (1/β ratio of 0.5) of supernatant were transferred into a 60 mL glass vial, via syringe with 0.20 μm filter pore. After the addition of 4 g of NaCl (≥99.5%, Sigma-Aldrich) and stirring bar of 20 × 5 mm, the vial was capped with a silicone/polytetrafluoroethylene septum and an aluminium cap (Chromacol Ltd., Herts, UK). The samples were stored at −80 °C until analysis.

The SPME holder for manual sampling and the coating fiber were purchased from Supelco (Aldrich, Bellefonte, PA, USA). The selected SPME device included a fused silica fiber coating, partially cross-linked with 50/30 μm divinylbenzene/carboxen™/polydimethylsiloxane StableFlex™ (1 cm), which comprehends a wide range capacity of sorbing compounds with different physicochemical properties^36^. After defrost, the vials were placed in a thermostated water bath so that headspace extraction was allowed to occur for 30 min, at 50 °C and under continuous agitation at 350 rpm. Three independent aliquots were analysed for each condition under study.

The SPME fiber was manually introduced into the GC × GC–ToFMS injection port and exposed during 30 seconds for thermal desorption into heated inlet (250 °C). The instrumental parameters were defined according to a previous study^48^. The inlet was lined with a 0.75 mm I.D. splitless glass liner and splitless injections mode were used (30 seconds). The LECO Pegasus 4D (LECO, St. Joseph, MI, USA) GC × GC–ToFMS system was comprised by an Agilent GC 7890A gas chromatograph (Agilent Technologies, Inc., Wilmington, DE), with a dual stage jet cryogenic modulator (licensed from Zoex) and a secondary oven, as well as mass spectrometer equipped with a ToF analyzer. An Equity-5 column (30 m × 0.32 mm I.D., 0.25 μm film thickness, Supelco, Inc., Bellefonte, PA, USA) and a DB-FFAP column (0.79 m × 0.25 mm I.D., 0.25 μm film thickness, J&W Scientific Inc., Folsom, CA, USA) were used for first (^1^D) and second (^2^D) dimensions, respectively. The carrier gas was helium at a constant flow rate of 2.50 mL min^−1^. The following temperature programs were used: the primary oven temperature was ranged from 40 °C (1 min) to 140 °C at 10 °C min^−1^, and then to 200 °C (1 min) at 7 °C min^−1^. The secondary oven temperature program was 15 °C offset above the primary oven. Both the MS transfer line and MS source temperatures were 250 °C. The modulation period was 5 seconds, keeping the modulator at 20 °C offset above primary oven, with hot and cold pulses by periods of 0.80 and 1.70 seconds, respectively. The ToF analyzer was operated at a spectrum storage rate of 100 spectra s^−1^, with mass spectrometer running in the EI mode at 70 eV and detector voltage of −1480 V, using an *m*/*z* range of 35–300. Total ion chromatograms were processed using the automated data processing software ChromaTOF^®^ (LECO) at signal-to-noise threshold of 200. For identification purposes, the mass spectrum and retention times (^1^D and ^2^D) of the analytes were compared with standards, when available. Also, the identification process was done by comparing the mass spectrum of each peak with existing ones in mass spectral libraries, which included an in-house library of standards and two commercial databases (Wiley 275 and US National Institute of Science and Technology (NIST) V. 2.0 - Mainlib and Replib). Moreover, a manual analysis of mass spectra was done, combining additional information like retention index (RI) value, which was experimentally determined according to van den Dool and Kratz RI equation^49^. A C_8_–C_20_ *n*-alkanes series was used for RI determination (the solvent *n*-hexane was used as C_6_ standard), comparing these values with reported ones in existing literature for chromatographic columns similar to ^1^D column above mentioned (Table S1). The majority (>90%) of the identified compounds presented similarity matches >800/1000. The Deconvoluted Total Ion Current GC × GC area data were used as an approach to estimate the relative content of each metabolite from fungi under study.

### Statistical analysis

A full data matrix from *A. niger* consisted of 24 observations (2 temperatures ×2 incubation periods ×2 growth media, each one by 3 independent assays) and 428 variables was constructed (Table S2). A heatmap visualization was applied for this dataset, normalizing each variable area by the maximum of each volatile compound for all samples (with GC peak previously normalized by CFU mL^−1^), using the Unscrambler^®^ × (30-day trial version – CAMO Software AS, Oslo, Norway).

In order to evaluate the influence of the main effects under study (culture medium, growth period and temperature, and their interactions (i.e. medium × temperature, medium × time, and temperature × time), an Analysis of Variance – Simultaneous Component Analysis (ASCA) was applied to the above dataset comprising 428 variables. Significance of each effect was assessed using a permutation test. Data were permuted 2000 times and the percentage of the variance explained by each sub-model in the total model was used as quality-of-fit criterion for the permutation test. Data were normalized by the total area and mean centred and auto-scaled prior to the calculations.

A sub-data set of 44 metabolites detected in all studied growth conditions was defined as *A. niger* molecular biomarkers pattern, and an exploratory test was done to evaluate its potential in fungi distinction. Prior, to multivariate analysis, the GC areas of the 44 selected analytes from *A. niger* cultures and respective medium control were compared. Other data matrix was constructed, consisting of 30 observations belonging to *C. albicans* and *P. chrysogenum*, each one consisting of 2 growth conditions (3 and 5 days, at 25 °C, in YGC_A_, each one with 3 independent assays), as well as to *A. niger*, also comprising the same conditions (3 and 5 days, at 25 °C, in YGC_A_, each one with 9 independent assays) (Table S3). The data were analysed by PLS-DA, with peak areas previously normalized by the total area, mean-centred and autoscaled, which is a data pre-treatment process that gives to variables the same weight. Dummy variables encoding class-membership were used as dependent variables in PLS-DA. Three dependent variables corresponding to three microorganisms were used. Scores scatter plots were used for visualizing the variability between samples. Variables were sorted according to VIP values, aiming to identify the metabolites that mostly contribute to the differentiation between the groups.

In order to reduce the data pre-processing time, a sub-data set of 16 metabolites with VIP value higher than 0.8 was selected (Table 2) to verify the potential usefulness of the developed methodology, which also underwent PLS-DA. The classification model complexity (number of latent variables) of the both sub-data sets was computed, as well as classification rate and Q^2^ (quality-of-fit criterion) were estimated by cross-validation. Model robustness was assessed using permutation test (1000 permutations). ASCA, PLS-DA, permutation test and VIP algorithms were conducted by MATLAB, v. 7.12.

## Additional Information

**How to cite this article**: Costa, C. P. *et al.* Shedding light on *Aspergillus niger *volatile exometabolome. *Sci. Rep.* **6**, 27441; doi: 10.1038/srep27441 (2016).

## Supplementary Material

Supplementary Information

Supplementary Dataset

## Figures and Tables

**Table 1:** Sub-data set of 44 metabolites for the A. niger molecular biomarkers pattern.

Peak number	^1^*t*_R_^a^ (s)	^2^*t*_R_^a^ (s)	Metabolite	CAS number	Formula	MSI level^b^	RI_Calc_^c^	RI_Lit_^d^	
								GC × GC	GC-MS
			**Alcohols**						
			*Aliphatic*						
14	115	0.910	1-Butanol	71-36-3	C_4_H_10_O	1	644	655	–
18	150	1.160	3-Methyl-1-butanol	123-51-3	C_5_H_12_O	1	718	706	–
27	255	1.140	1-Hexanol	111-27-3	C_6_H_14_O	1	878	877	–
37	345	1.100	1-Heptanol	111-70-6	C_7_H_16_O	2	975	974	–
39	350	1.050	1-Octen-3-ol	3391-86-4	C_8_H_16_O	1	980	992	–
44	365	1.270	3-Octanol	589-98-0	C_8_H_18_O	1	996	–	996
46	395	0.990	2-Ethyl-1-hexanol	104-76-7	C_8_H_18_O	2	1029	1038	–
51	440	1.030	1-Octanol	111-87-5	C_8_H_18_O	1	1079	1079	–
			*Aromatic*						
76	475	3.030	2-Phenylethanol	60-12-8	C_8_H_10_O	1	1120	1107	–
80	805	2.060	2,4-bis (1,1-Dimethylethyl)phenol	96-76-4	C_14_H_22_O	2	1514	–	1513
			**Aldehydes**						
			*Aliphatic*						
83	110	0.460	3-Methylbutanal	590-86-3	C_5_H_10_O	2	633	628	–
92	190	0.590	Hexanal	66-25-1	C_6_H_12_O	1	801	800	–
94	275	0.620	Heptanal	111-71-7	C_7_H_14_O	1	901	903	–
104	465	0.630	Nonanal	124-19-6	C_9_H_18_O	1	1106	1106	–
107	555	0.630	Decanal	112-31-2	C_10_H_20_O	2	1207	1206	–
113	685	0.770	2-Undecenal	2463-77-6	C_11_H_20_O	2	1364	–	1376
114	720	0.650	Dodecanal	112-54-9	C_12_H_24_O	2	1407	1406	–
			*Aromatic*						
118	335	1.550	Benzaldehyde	100-52-7	C_7_H_6_O	1	965	964	–
119	410	1.620	Benzeneacetaldehyde	122-78-1	C_8_H_8_O	1	1046	1049	–
			**Esters**						
			*Aliphatic*						
131	135	0.530	Methyl 2-methylpropenoate	80-62-6	C_5_H_8_O_2_	2	685	710	–
168	695	0.920	3-Hydroxy-2,4,4-trimethylpentyl 2-methylpropanoate	74367-34-3	C_12_H_24_O_3_	2	1376	–	1381
			*Aromatic*						
184	545	1.050	2-Phenylethylacetate	103-45-7	C_10_H_12_O_2_	2	1196	–	1192
			**Hydrocarbons**						
			*Aliphatic*						
232	880	0.490	Hexadecane	544-76-3	C_16_H_34_	1	1601	1600	–
233	965	0.430	Heptadecane	629-78-7	C_17_H_36_	1	1701	1700	–
			*Aromatic*						
236	115	0.460	Benzene	71-43-2	C_6_H_6_	1	643	–	648
237	170	0.540	Toluene	108-88-3	C_7_H_8_	1	759	771	–
239	250	0.590	1,3-Dimethylbenzene	108-38-3	C_8_H_10_	2	871	–	874
240	270	0.640	1,2-Dimethylbenzene	95-47-6	C_8_H_10_	2	901	900	–
243	325	0.580	Propylbenzene	103-65-1	C_9_H_12_	2	953	959	–
245	335	0.590	1-Ethyl-4-methylbenzene	622-96-8	C_9_H_12_	2	964	970	–
248	365	0.640	1,3,5-Trimethylbenzene	108-67-8	C_9_H_12_	2	995	974	–
250	390	0.580	1-Methyl-2-(1-methylethyl)benzene	527-84-4	C_10_H_14_	2	1023	–	1022
251	390	0.690	1,2,3-Trimethylbenzene	526-73-8	C_9_H_12_	1	1023	–	1022
253	425	0.610	2-Ethyl-1,4-dimethylbenzene	1758-88-9	C_10_H_14_	2	1062	–	1087
263	700	1.270	Biphenyl	92-52-4	C_12_H_10_	2	1383	–	1385
265	880	1.020	2-Methyl-6-phenyl-1,6-heptadiene	51708-97-5	C_14_H_18_	2	1601	–	–
			**Ketones**						
			*Aliphatic*						
284	75	0.390	2-Propanone	67-64-1	C_3_H_6_O	1	559	–	503
297	265	0.580	3-Heptanone	106-35-4	C_7_H_14_O	1	889	884	–
302	355	0.740	6-Methyl-5-hepten-2-one	110-93-0	C_8_H_14_O	1	985	985	–
313	495	0.760	3-Nonen-2-one	18402-83-0	C_9_H_16_O	2	1140	–	1144
331	755	0.800	6,10-Dimethyl-5,9-undecadien-2-one	3796-70-1	C_13_H_22_O	2	1451	–	1455
			**Monoterpenenic compound**						
289	435	0.790	2,6-Dimethyl-7-octen-2-ol	18479-58-8	C_10_H_20_O	2	1073	–	1075
408	625	0.630	Endobornyl acetate	76-49-3	C_12_H_20_O_2_	2	1289	–	1285
			**Norisoprenoid**						
428	780	0.750	α-Methylionone	127-51-5	C_14_H_22_O	2	1482	–	1481

^a^Retention times for first (^1^*t*_R_) and second (^2^*t*_R_) dimensions in seconds.

^b^Level of metabolite identification1. (1) Identified compounds; (2) Putatively annotated compounds; (3) Putatively characterized compound classes; (4) Unknown compounds.

^c^RI_Calc_: Retention Index obtained through the modulated chromatogram.

^d^RI_Lit_: Retention Index reported in the literature for Equity-5 column or equivalents (see references in Table S1, from Supplementary Information).

**Table 2:** Sub-data set of the 16 metabolites with VIP (Variable importance in Projection) value higher than 0.8.

Peak number	Metabolites	VIP values
114	Dodecanal	1.32
104	Nonanal	1.31
51	1-Octanol	1.25
46	2-Ethyl-1-hexanol	1.23
107	Decanal	1.18
44	3-Octanol	1.16
297	3-Heptanone	1.04
428	α-Methyl ionone	1.04
168	3-Hydroxy-2,4,4-trimethylpentyl2-methyl-propanoate	1.04
118	Benzaldehyde	0.96
76	2-Phenylethanol	0.94
39	1-Octen-3-ol	0.86
37	1-Heptanol	0.85
284	2-Propanone	0.84
232	Hexadecane	0.82
18	3-Methyl-1-butanol	0.81
