# Supplementary material for: Shedding light on Aspergillus niger volatile exometabolome
Source: Sci Rep. 2016 Jun 6;6:27441. doi: 10.1038/srep27441 (PMC4893740; doi:10.1038/srep27441)
Supplement: Supplementary Information [file srep27441-s1.pdf]

Supplementary information

Shedding light on *Aspergillus niger* volatile exometabolome

Carina Pedrosa Costa, Diogo Gonçalves Silva, Alisa Rudnitskaya, Adelaide Almeida* & Sílvia M. Rocha*

* corresponding authors

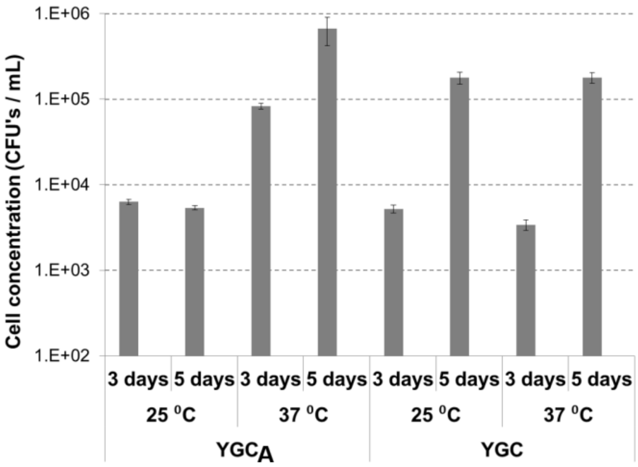

Figure S1. Cell concentration of *A. niger* cultures. Cell concentration (CFU mL^{-1}) of *A. niger* cultures in different growth conditions: medium (YGC_A and YGC), temperature (25 and 37 °C) and incubation period (3 and 5 days).

.

Table S1. Chromatographic data of the 428 analytes putatively identified from *A. niger* cultures, considering all the conditions under study, using HS-SPME/GC×GC-ToFMS. The metabolites assigned as molecular biomarkers pattern of *A. niger* are highlighted in bold.

[a] Retention times for first ($^{1}t_{R}$) and second ($^{2}t_{R}$) dimensions in seconds.
[b] Level of metabolite identification [1]. (1) Identified compounds; (2) Putatively annotated compounds; (3) Putatively characterized compound classes; (4) Unknown compounds.
[c] RI_{Calc}: Retention Index obtained through the modulated chromatogram.
[d] RI_{Lit}: Retention Index reported in the literature for Equity-5 column or equivalents.

Peak number	$^{1}t_{R}$[a] (s)	$^{2}t_{R}$[a] (s)	Metabolite	CAS number	Formula	MSI Level[b]	RI_{Calc}[c]	RI_{Lit}[d]		
								GC×GC	GC-MS	Ref.
			**Acids**							
			Aliphatic							
1	185	0.550	2-Methylpropanoic acid	79-31-2	$C_4H_8O_2$	1	791	-	775	[2]
2	200	1.860	Butanoic acid	107-92-6	$C_4H_8O_2$	1	814	-	821	[3]
3	240	1.130	3-Methylbutanoic acid	503-74-2	$C_5H_{10}O_2$	1	860	-	864	[4]
4	255	0.410	2-Methylbutanoic acid	116-53-0	$C_5H_{10}O_2$	2	877	-	873	[3]
5	485	3.370	2-Ethylhexanoic acid	149-57-5	$C_8H_{16}O_2$	2	1132	-	1129	[5]
6	535	3.680	Octanoic acid	124-07-2	$C_8H_{16}O_2$	1	1188	-	1182	[6]
7	615	3.200	Nonanoic acid	112-05-0	$C_9H_{18}O_2$	1	1280	-	1280	[5]
8	695	2.890	Decanoic acid	334-48-5	$C_{10}H_{20}O_2$	2	1379	-	1380	[7]
9	1020	2.650	Tetradecanoic acid	544-63-8	$C_{14}H_{28}O_2$	2	1774	-	1780	[7]
10	1105	2.590	Pentadecanoic acid	1002-84-2	$C_{15}H_{30}O_2$	2	1868	-	1878	[7]
			**Alcohols**							
			Aliphatic							
11	85	0.700	1-Propanol	71-23-8	C_3H_8O	1	580	-	574	[8]
12	95	0.640	2-Butanol	78-92-2	$C_4H_{10}O$	1	601	603	-	[9]
13	100	0.800	2-Methyl-1-propanol	78-83-1	$C_4H_{10}O$	1	612	615	-	[10]
**14**	**115**	**0.910**	**1-Butanol**	**71-36-3**	$\mathbf{C_4H_{10}O}$	**1**	**644**	**655**	**-**	[9]
15	130	0.740	3-Pentanol	584-02-1	$C_5H_{12}O$	2	675	-	710	[7]
16	130	0.770	2-Pentanol	6032-29-7	$C_5H_{12}O$	2	675	-	685	[11]
17	145	1.260	3-Methyl-3-buten-1-ol	763-32-6	$C_5H_{10}O$	1	708	-	716	[12]
**18**	**150**	**1.160**	**3-Methyl-1-butanol**	**123-51-3**	$\mathbf{C_5H_{12}O}$	**1**	**718**	**706**	**-**	[10]
19	165	0.800	4-Methyl-2-pentanol	108-11-2	$C_6H_{14}O$	2	749	-	758	[13]
20	170	0.770	2-Methyl-3-pentanol	565-67-3	$C_6H_{14}O$	2	760	-	774	[14]
21	170	1.120	1-Pentanol	71-41-0	$C_5H_{12}O$	1	760	776	-	[15]
22	180	1.490	3-Methyl-2-buten-1-ol	556-82-1	$C_5H_{10}O$	2	782	-	778	[5]
23	185	0.860	3-Methyl-2-pentanol	565-60-6	$C_6H_{14}O$	2	791	-	-	-
24	190	0.820	3-Hexanol	623-37-0	$C_6H_{14}O$	2	801	-	806	[16]
25	225	1.130	4-Methyl-1-pentanol	626-89-1	$C_6H_{14}O$	2	843	-	846	[17]

26	245	1.400	4-Methyl-3-penten-1-ol	763-89-3	$C_6H_{12}O$	2	866	-	-	-
**27**	**255**	**1.140**	**1-Hexanol**	**111-27-3**	$C_6H_{14}O$	**1**	**878**	**877**	**-**	[10]
28	255	1.480	4-Hexen-1-ol	928-92-7	$C_6H_{12}O$	2	878	-	883	[18]
29	260	1.100	1-Hepten-3-ol	4938-52-7	$C_7H_{14}O$	2	884	-	881	[19]
30	270	0.820	4-Heptanol	589-55-9	$C_7H_{16}O$	2	895	-	879	[20]
31	275	0.840	3-Heptanol	589-82-2	$C_7H_{16}O$	2	901	892	-	[9]
32	280	0.890	2-Heptanol	543-49-7	$C_7H_{16}O$	2	906	-	904	[8]
33	310	1.140	2-Hepten-1-ol (isomer)	22104-77-4	$C_7H_{14}O$	2	938	-	-	-
34	315	0.840	Butoxypropanol	5131-66-8	$C_7H_{16}O_2$	2	943	-	947	[21]
35	330	1.150	5-Methyl-1-hepten-4-ol	99328-46-8	$C_8H_{16}O$	2	959	-	-	-
36	335	0.870	2-Octanol	123-96-6	$C_8H_{18}O$	2	964	-	962	[22]
**37**	**345**	**1.100**	**1-Heptanol**	**111-70-6**	$C_7H_{16}O$	**1**	**975**	**974**	**-**	[15]
38	345	1.380	2-Hepten-1-ol (isomer)	22104-77-4	$C_7H_{14}O$	2	975	-	970	[8]
**39**	**350**	**1.050**	**1-Octen-3-ol**	**3391-86-4**	$C_8H_{16}O$	**1**	**980**	**992**	**-**	[23]
40	350	1.210	Octa-1,5-dien-3-ol	50306-18-8	$C_8H_{14}O$	2	980	-	-	-
41	355	0.930	5-Octen-3-ol		$C_8H_{16}O$	2	985	-	-	-
42	365	1.010	6-Methyl-1-heptanol	1653-40-3	$C_8H_{18}O$	2	996	-	-	-
43	365	1.030	6-Methyl-5-hepten-2-ol	1569-60-4	$C_8H_{16}O$	2	996	-	995	[18]
**44**	**365**	**1.270**	**3-Octanol**	**589-98-0**	$C_8H_{18}O$	**1**	**996**	**-**	**996**	[24]
45	390	1.100	3-Ethyl-4-methyl-1-pentanol	100431-87-6	$C_8H_{18}O$	2	1023	-	-	-
**46**	**395**	**0.990**	**2-Ethyl- 1-hexanol**	**104-76-7**	$C_8H_{18}O$	**2**	**1029**	**1038**	**-**	[23]
47	405	1.040	4-Methyl-1-heptanol	817-91-4	$C_8H_{18}O$	2	1040	-	-	-
48	415	1.030	5-Methyl-1-heptanol	7212-53-5	$C_8H_{18}O$	2	1051	-	-	-
49	435	1.280	2-Octen-1-ol	18409-17-1	$C_8H_{16}O$	2	1074	-	1066	[25]
50	440	0.740	1-Nonen-3-ol	21964-44-3	$C_9H_{18}O$	2	1079	-	1079	[19]
**51**	**440**	**1.030**	**1-Octanol**	**111-87-5**	$C_8H_{18}O$	**1**	**1079**	**1079**	**-**	[23]
52	460	0.840	2-Nonanol	628-99-9	$C_9H_{20}O$	2	1101	-	1098	[5]
53	465	0.990	7-Octen-2-ol	39546-75-3	$C_8H_{16}O$	2	1107	-	-	-
54	480	1.030	3-Octen-1-ol	18185-81-4	$C_8H_{16}O$	2	1123	-	-	-
55	490	1.010	1-Octen-4-ol	40575-42-6	$C_8H_{16}O$	2	1135	-	-	-
56	505	1.040	2-Nonen-1-ol (isomer)	22104-79-6	$C_9H_{18}O$	2	1151	1074	-	[26]
57	525	0.970	1-Nonanol	143-08-8	$C_9H_{20}O$	2	1173	1179	-	[23]
58	530	1.110	2-Nonen-1-ol (isomer)	22104-79-6	$C_9H_{18}O$	2	1179	-	-	-
59	545	0.760	3-Decanol	1565-81-7	$C_{10}H_{22}O$	2	1195	-	1188	[5]
60	545	1.360	2-(2-Butoxyethoxy)ethanol	112-34-5	$C_8H_{18}O_3$	2	1196	1192	-	[27]
61	565	0.860	2-Propyl-1-heptanol	10042-59-8	$C_{10}H_{22}O$	2	1219	-	-	-
62	615	0.620	2-Decen-1-ol	18409-18-2	$C_{10}H_{20}O$	2	1277	1281	-	[28]
63	615	0.910	1-Decanol	112-30-1	$C_{10}H_{22}O$	1	1278	1281	-	[23]
64	625	0.730	4-Undecanol	4272-06-4	$C_{11}H_{24}O$	2	1289	-	1281	[20]
65	630	0.700	2,4-Undecadien-1-ol	77657-78-4	$C_{11}H_{20}O$	2	1295	-	-	-
66	635	0.770	2-Undecanol	1653-30-1	$C_{11}H_{24}O$	1	1301	-	1303	[29]
67	645	0.830	2-Butyl-1-octanol	3913-02-8	$C_{12}H_{26}O$	2	1314	-	-	-
68	725	0.860	2-Dodecanol	10203-28-8	$C_{12}H_{26}O$	1	1414	1413	-	[23]

69	760	0.960	6,10-Dimethyl-5,9-undecadien-2-ol	53837-34-6	$C_{13}H_{24}O$	2	1458	-	1459	[30]
70	775	0.940	1-Dodecanol	112-53-8	$C_{12}H_{26}O$	1	1476	1480	-	[23]
71	950	0.970	1-Tetradecanol	112-72-1	$C_{14}H_{30}O$	2	1684	1686	-	[23]
			Aromatic							
72	410	4.130	Benzylalcohol	100-51-6	C_7H_8O	1	1049	1044	-	[31]
73	430	2.770	Methylbenzenemethanol	1445-91-6	$C_8H_{10}O$	2	1070	-	-	-
74	450	1.990	2-Phenylisopropanol	617-94-7	$C_9H_{12}O$	2	1091	1080	-	[32]
75	455	1.070	4-Ethyl-1,3-benzenediol	2896-60-8	$C_8H_{10}O_2$	2	1096	-	-	-
**76**	**475**	**3.030**	**2-Phenylethanol**	**60-12-8**	$\mathbf{C_8H_{10}O}$	**1**	**1120**	**1107**	**-**	[33]
77	495	1.940	Methylbenzeneethanol	698-87-3	$C_9H_{12}O$	2	1141	-	-	-
78	555	2.590	2,4,6-Trimethylphenol	527-60-6	$C_9H_{12}O$	2	1209	-	1203	[34]
79	680	2.700	2-(1,1-Dimethylethyl)-4-methylphenol	2409-55-4	$C_{11}H_{16}O$	2	1360	-	1387	[35]
**80**	**805**	**2.060**	**2,4-bis(1,1-Dimethylethyl)phenol**	**96-76-4**	$\mathbf{C_{14}H_{22}O}$	**2**	**1514**	**-**	**1513**	[24]
81	810	0.780	Butylhydroxytoluene	128-37-0	$C_{15}H_{24}O$	2	1519	-	1518	[24]
82	890	3.240	1,1,3,3-Tetramethylbutylphenol	27193-28-8	$C_{14}H_{22}O$	2	1616	-	-	-
			Cyclic							
83	165	1.470	Cyclopropaneethanol	2566-44-1	$C_5H_{10}O$	2	750	-	-	-
84	545	1.150	3-Methylcyclohexanol	591-23-1	$C_7H_{14}O$	2	1196	-	-	-
			**Aldehydes**							
			Aliphatic							
85	70	0.340	Acetaldehyde	75-07-0	C_2H_4O	1	548	-	500	[36]
**86**	**110**	**0.460**	**3-Methylbutanal**	**590-86-3**	$\mathbf{C_5H_{10}O}$	**1**	**633**	**628**	**-**	[37]
87	110	0.650	2-Butenal	4170-30-3	C_4H_6O	2	633	657	-	[27]
88	115	0.440	2-Methylbutanal	96-17-3	$C_5H_{10}O$	2	643	635	-	[37]
89	120	0.490	*m/z* 55, 84, 39, 56	-	C_5H_8O	3	654	-	-	-
90	125	0.500	Pentanal	110-62-3	$C_5H_{10}O$	1	664	691	-	[9]
91	155	0.670	2-Methyl-2-butenal	497-03-0	C_5H_8O	2	728	-	739	[38]
**92**	**190**	**0.590**	**Hexanal**	**66-25-1**	$\mathbf{C_6H_{12}O}$	**1**	**801**	**800**	**-**	[39]
93	195	0.710	3-Hexenal	6789-80-6	$C_6H_{10}O$	2	807	-	801	[40]
**94**	**275**	**0.620**	**Heptanal**	**111-71-7**	$\mathbf{C_7H_{14}O}$	**1**	**901**	**903**	**-**	[39]
95	325	0.560	2-Ethylhexanal	123-05-7	$C_8H_{16}O$	2	953	955	-	[9]
96	330	0.790	2-Heptenal	18829-55-5	$C_7H_{12}O$	2	959	956	-	[9]
97	360	0.680	5-Methyl-2-heptenal	94705-03-0	$C_8H_{14}O$	2	990	-	-	-
98	370	0.640	Octanal	124-13-0	$C_8H_{16}O$	1	1001	1004	-	[39]
99	415	0.740	2-Octenal (isomer)	2363-89-5	$C_8H_{14}O$	2	1051	1056	-	[32]
100	420	0.650	2,6-Dimethyl-5-heptenal	106-72-9	$C_9H_{16}O$	2	1056	-	1060	[41]
101	425	0.780	2-Octenal (isomer)	2363-89-5	$C_8H_{14}O$	2	1062	1056	-	[15]
102	425	0.910	2,6-Octadienal	76917-23-2	$C_8H_{12}O$	2	1062	-	-	-
103	430	0.640	2-Methylenehexanal	1070-66-2	$C_7H_{12}O$	2	1067	-	-	-
**104**	**465**	**0.630**	**Nonanal**	**124-19-6**	$\mathbf{C_9H_{18}O}$	**1**	**1106**	**1106**	**-**	[10]
105	515	0.770	2-Nonenal	18829-56-6	$C_9H_{16}O$	2	1162	-	1164	[13]

106	545	0.700	4-Decenal	21662-09-9	$C_{10}H_{18}O$	2	1195	-	1193	[42]
**107**	**555**	**0.630**	**Decanal**	**112-31-2**	$\mathbf{C_{10}H_{20}O}$	**1**	**1207**	**1206**	**-**	[39]
108	565	0.990	2,4-Nonadienal	6750-03-4	$C_9H_{14}O$	2	1219	-	1212	[43]
109	600	0.770	2-Decenal	2497-25-8	$C_{10}H_{18}O$	2	1260	-	1261	[5]
110	630	0.920	2,4-Decadienal (isomer)	2363-88-4	$C_{10}H_{16}O$	2	1295	-	1298	[44]
111	640	0.620	Undecanal	112-44-7	$C_{11}H_{22}O$	2	1307	1306	-	[39]
112	650	0.960	2,4-Decadienal (isomer)	2363-88-4	$C_{10}H_{16}O$	2	1320	-	1314	[42]
**113**	**685**	**0.770**	**2-Undecenal**	**2463-77-6**	$\mathbf{C_{11}H_{20}O}$	**2**	**1364**	**-**	**1376**	[45]
**114**	**720**	**0.650**	**Dodecanal**	**112-54-9**	$\mathbf{C_{12}H_{24}O}$	**1**	**1407**	**1406**	**-**	[39]
115	730	1.010	2,4-Undecadienal	30361-29-6	$C_{11}H_{18}O$	2	1420	-	1416	[46]
116	805	0.680	Tridecanal	10486-19-8	$C_{13}H_{26}O$	2	1513	1512	-	[39]
117	890	0.710	Tetradecanal	124-25-4	$C_{14}H_{28}O$	2	1613	1613	-	[39]
			Aromatic							
**118**	**335**	**1.550**	**Benzaldehyde**	**100-52-7**	$\mathbf{C_7H_6O}$	**1**	**965**	**964**	**-**	[26]
**119**	**410**	**1.620**	**Benzeneacetaldehyde**	**122-78-1**	$\mathbf{C_8H_8O}$	**1**	**1046**	**1049**	**-**	[9]
120	410	1.860	2-Hydroxybenzaldehyde	90-02-8	$C_7H_6O_2$	2	1047	-	1041	[5]
121	435	1.360	2-Methylbenzaldehyde	529-20-4	C_8H_8O	2	1074	-	1067	[7]
122	445	1.410	4-Methylbenzaldehyde	104-87-0	C_8H_8O	2	1085	-	1079	[7]
123	515	1.640	2-Phenylpropenal	4432-63-7	C_9H_8O	2	1163	-	1148	[47]
124	565	1.360	3,5-Dimethylbenzaldehyde	5779-95-3	$C_9H_{10}O$	2	1219	-	-	-
125	585	1.140	4-(1-Methylethyl)-benzaldehyde	122-03-2	$C_{10}H_{12}O$	2	1243	-	1242	[48]
126	620	2.060	3-Phenyl-2-propenal	104-55-2	C_9H_8O	2	1285	1296	-	[49]
127	640	1.090	4-Butylbenzaldehyde	939-97-9	$C_{11}H_{14}O$	2	1308	-	-	-
128	820	1.030	Lily aldehyde	-	$C_{14}H_{20}O$	2	1531	-	1532	[50]
			**Esters**							
			Aliphatic							
129	95	0.440	Ethyl acetate	141-78-6	$C_4H_8O_2$	1	601	-	608	[51]
130	130	0.510	Ethyl propenoate	140-88-5	$C_5H_8O_2$	1	675	-	702	[52]
**131**	**135**	**0.530**	**Methyl 2-methylpropenoate**	**80-62-6**	$\mathbf{C_5H_8O_2}$	**2**	**685**	**710**	**-**	[9]
132	165	0.460	Ethyl isobutyrate	97-62-1	$C_6H_{12}O_2$	2	748	-	747	[53]
133	175	0.500	Isobutyl ethanoate	110-19-0	$C_6H_{12}O_2$	2	769	-	770	[11]
134	195	0.520	Ethyl butanoate	105-54-4	$C_6H_{12}O_2$	2	806	-	800	[5]
135	205	0.550	Butyl ethanoate	123-86-4	$C_6H_{12}O_2$	2	818	-	812	[54]
136	235	0.510	2-Pentyl acetate	626-38-0	$C_7H_{14}O_2$	2	854	-	-	-
137	255	0.550	Isoamyl ethanoate	123-92-2	$C_7H_{14}O_2$	2	877	-	876	[54]
138	255	0.730	1-Methoxy-2-propyl acetate	108-65-6	$C_6H_{12}O_3$	2	877	-	-	-
139	260	0.540	2-Methylbutyl acetate	624-41-9	$C_7H_{14}O_2$	2	883	-	880	[7]
140	315	0.640	Ethyl tiglate	5837-78-5	$C_7H_{12}O_2$	2	943	-	949	[7]
141	320	1.340	2-Methylamyl acetate	7789-99-3	$C_9H_{20}O$	2	949	-	-	-
142	330	0.510	Isobutyl butanoate	539-90-2	$C_8H_{16}O_2$	2	958	-	958	[55]
143	340	0.700	*m/z* 43, 71, 87, 59	-		3	969	-	-	-

144	365	0.540	Butyl butanoate	109-21-7	$C_8H_{16}O_2$	2	995	-	993	[54]
145	365	0.620	3-Methylbutyl-2-propenoate	-	$C_8H_{14}O_2$	2	995	-	-	-
146	370	1.420	Ethylene diethanoate	111-55-7	$C_6H_{10}O_4$	2	1002	-	-	-
147	370	0.560	Ethyl hexanoate	123-66-0	$C_8H_{16}O_2$	1	1001	-	997	[13]
148	375	0.440	Propyl pivalate	5129-35-1	$C_8H_{16}O_2$	2	1006	-	-	-
149	375	0.550	Pentyl propanate	624-54-4	$C_8H_{16}O_2$	2	1006	-	1006	[56]
150	385	1.100	Ethyl 2-methyl-3-oxopentanoate	17422-12-7	$C_7H_{12}O_3$	2	1018	-	-	-
151	410	0.530	Methyl 2-ethylhexanoate	816-19-3	$C_9H_{18}O_2$	2	1045	-	1043	[57]
152	420	0.530	Isopentyl butanoate	106-27-4	$C_9H_{18}O_2$	2	1056	-	1054	[54]
153	440	0.560	*t*-Butyl acetoacetate	1694-31-1	$C_8H_{14}O_3$	2	1078	-	-	-
154	455	0.740	2-Butoxyethyl acetate	112-07-2	$C_8H_{16}O_3$	2	1095	-	1096	[58]
155	460	0.550	Ethyl heptanoate	106-30-9	$C_9H_{18}O_2$	2	1101	-	1095	[5]
156	510	0.550	3-Methylheptyl acetate	72218-58-7	$C_{10}H_{20}O_2$	2	1156	-	-	-
157	520	1.230	3-Methylphenyl acetate	122-46-3	$C_9H_{10}O_2$	2	1168	-	-	-
158	545	0.560	Ethyl octanoate	106-32-1	$C_{10}H_{20}O_2$	1	1195	-	1195	[5]
159	560	0.920	Dimethyl 2,4-dimethylpentanedioate	2121-68-8	$C_9H_{16}O_4$	2	1213	-	-	-
160	575	0.580	2-Ethylhexyl 2-propenoate	103-11-7	$C_{11}H_{20}O_2$	2	1230	-	-	-
161	620	0.970	Methyl 2-phenylbutanoate	2294-71-5	$C_{11}H_{14}O_2$	2	1284	-	-	-
162	630	0.560	Ethyl nonanoate	123-29-5	$C_{11}H_{22}O_2$	1	1295	-	1294	[7]
163	630	0.600	4-*tert*-Butylcyclohexyl acetate (isomer)	32210-23-4	$C_{12}H_{22}O_2$	2	1295	-	-	-
164	660	0.630	4-*tert*-Butylcyclohexyl acetate (isomer)	32210-23-4	$C_{12}H_{22}O_2$	2	1332	-	-	-
165	665	1.120	Methyl 2-(phenylmethyl)prop-2-enoate	3070-71-1	$C_{11}H_{12}O_2$	2	1339	-	-	-
166	675	0.750	3-Phenyl-2-propenyl propionate	103-56-0	$C_{12}H_{14}O_2$	2	1351	-	-	-
167	690	0.650	4-*tert*-Butylcyclohexyl acetate (isomer)	32210-23-4	$C_{12}H_{22}O_2$	2	1370	-	1368	[59]
**168**	**695**	**0.920**	**3-Hydroxy-2,4,4-trimethylpentyl 2-methyl-propanoate**	**74367-34-3**	$\mathbf{C_{12}H_{24}O_3}$	**2**	**1376**	**-**	**1381**	[32]
169	710	0.580	Ethyl decanoate	110-38-3	$C_{12}H_{24}O_2$	1	1395	-	1394	[5]
170	725	0.860	Decyl 2-methoxyacetate	259141-02-1	$C_{13}H_{26}O_3$	2	1414	-	-	-
171	760	0.970	Dibutyl-2-butenedioate	105-76-0	$C_{12}H_{20}O_4$	2	1458	-	-	-
172	760	1.080	Ethyl 2-phenylbutanoate	119-43-7	$C_{12}H_{16}O_2$	2	1458	-	-	-
173	775	0.840	Diisobutyl butanedioate	925-06-4	$C_{12}H_{22}O_4$	2	1476	-	-	-
174	790	0.830	Dimethylphenethyl butyrate	10094-34-5	$C_{14}H_{20}O_2$	2	1495	-	1488	[60]
175	800	1.090	Ethyl 5-phenyl-2-pentenoate	55282-95-6	$C_{13}H_{16}O_2$	2	1507	-	-	-
176	905	0.600	Isopropyl dodecanoate	10233-13-3	$C_{15}H_{30}O_2$	2	1630	-	1618	[61]
177	960	0.550	3-Tridecanyl propionate	-	$C_{16}H_{32}O_2$	2	1695	-	-	-
178	985	0.650	4-Tridecanyl propionate	-	$C_{16}H_{32}O_2$	2	1724	-	-	-
179	1070	0.660	Isopropyl tetradecanoate	110-27-0	$C_{17}H_{34}$O2	2	1824	1801	-	[23]
			Aromatic							
180	460	1.210	Methyl benzoate	93-58-3	$C_8H_8O_2$	1	1101	-	1091	[5]
181	520	1.270	Benzyl ethanoate	140-11-4	$C_9H_{10}O_2$	2	1168	-	1163	[5]
182	525	1.050	Ethyl benzoate	93-89-0	$C_9H_{10}O_2$	1	1173	-	1170	[5]
183	545	1.050	Phenylethyl acetate	93-92-5	$C_{10}H_{12}O_2$	1	1196	-	1192	[5]

**184**	**600**	**1.160**	**2-Phenylethyl acetate**	**103-45-7**	**$C_{10}H_{12}O_2$**	**2**	**1260**	**-**	**1256**	[5]
185	615	1.190	Methyl benzenepropanoate	103-25-3	$C_{10}H_{12}O_2$	2	1278	-	1280	[62]
186	640	1.020	Phenylpropyl acetate	10402-52-5	$C_{11}H_{14}O_2$	2	1308	-	-	-
187	650	0.860	Dimethylphenethyl acetate	151-05-3	$C_{12}H_{16}O_2$	2	1320	-	-	-
188	655	0.910	Isobutyl benzoate	120-50-3	$C_{11}H_{14}O_2$	2	1326	-	1346	[63]
189	675	0.750	3-Phenyl-2-propenyl propionate	103-56-0	$C_{12}H_{14}O_2$	2	1351	-	-	-
190	695	0.970	4-Phenyl-2-butyl acetate	10415-88-0	$C_{12}H_{16}O_2$	2	1376	-	1398	[64]
191	710	0.940	o-Methylbenzyl acetate	17373-93-2	$C_{10}H_{12}O_2$	2	1395	-	-	-
192	730	1.040	Methyl 2-methyl-4-phenyl butanoate	-	$C_{12}H_{16}O_2$	2	1420	-	-	-
193	790	0.820	Dimethyl benzyl carbinyl butyrate	10094-34-5	$C_{14}H_{20}O_2$	2	1495	-	1488	[60]
194	790	1.220	Methyl 5-phenylvalerate	20620-59-1	$C_{12}H_{16}O_2$	2	1495	-	-	-
195	795	1.110	Ethyl-5-phenyl-2-pentenoate	55282-95-6	$C_{13}H_{16}O_2$	2	1501	-	-	-
196	860	1.090	Amyl salicylate	2050-08-0	$C_{12}H_{16}O_3$	1	1578	-	-	-
			**Ethers**							
			Aliphatic							
197	120	0.470	2-Ethoxybutane	2679-87-0	$C_6H_{14}O$	2	654	-	622	[18]
198	330	0.680	Vinyl (2-butoxy)ethyl ether	4223-11-4	$C_8H_{16}O_2$	2	959	-	-	-
199	435	0.530	Methoxycyclohexane	931-56-6	$C_7H_{14}O$	2	1073	-	-	-
200	725	0.860	1-(Methoxymethoxy)hexane	66675-06-7	$C_8H_{18}O_2$	2	1414	-	-	-
			Aromatic							
201	390	0.910	1-Methoxy-3-methyl-benzene	100-84-5	$C_8H_{10}O$	2	1023	-	1028	[65]
202	445	0.860	2-Methoxyethylbenzene	3558-60-9	$C_9H_{12}O$	2	1084	-	1080	[5]
203	520	1.320	1,4-Dimethoxybenzene	150-78-7	$C_8H_{10}O_2$	2	1168	-	1163	[5]
204	535	0.710	(1,1-Dimethylethoxy)methylbenzene	3459-80-1	$C_{11}H_{16}O$	2	1184	-	-	-
205	550	0.960	1-Methoxy-4-(2-propenyl)benzene	140-67-0	$C_{10}H_{12}O$	2	1201	-	1195	[42]
206	715	1.270	1,1-Oxybis-benzene	101-84-8	$C_{12}H_{10}O$	2	1402	-	1396	[7]
207	755	1.040	Dimethoxymethyl-*tert*-butylbenzene	-	$C_{13}H_{20}O_2$	2	1451	-	-	-
208	805	1.750	1-Methoxy-4-(4-methyl-4-pentenyl)benzene	74672-06-3	$C_{13}H_{18}O$	2	1514	-	-	-
			**Hydrocarbons**							
			Aliphatic							
209	130	0.340	Heptane	142-82-5	C_7H_{16}	1	674	-	700	[5]
210	190	0.360	2,4-Dimethylheptane	2213-23-2	C_9H_{20}	2	800	819	-	[10]
211	215	0.420	1,3-Octadiene	1002-33-1	C_8H_{14}	2	830	-	827	[13]
212	275	0.370	Nonane	111-84-2	C_9H_{20}	1	900	900	-	[26]
213	365	0.370	C10 (*m/z* 57, 41, 39, 55)	-	-	3	995	-	-	-
214	370	0.380	Decane	124-18-5	$C_{10}H_{22}$	1	1000	1000	-	[39]
215	375	0.380	C10 (*m/z* 57, 97, 41, 55)	123-48-8	$C_{12}H_{24}$	3	1006	-	-	-
216	400	0.390	3,4,4-Trimethyl-2-pentene	598-96-9	C_8H_{16}	2	1034	-	-	-
217	410	0.390	2,3,4-Trimethyl-2-pentene	565-77-5	C_8H_{16}	2	1045	-	-	-

218	410	0.710	Nonatetra-1,3,5,7-ene	83829-35-0	C_9H_{12}	2	1045	-	-	-
219	420	0.400	C10 (*m/z* 57, 41, 55, 69)	7756-94-7	$C_{12}H_{24}$	3	1056	-	-	-
220	420	0.740	C10 (*m/z* 41, 69, 39, 67)	998-94-7	C_8H_{14}	3	1056	-	-	-
221	425	1.060	C10 (*m/z* 41, 70, 39, 43)	16106-59-5	C_8H_{16}	3	1062	-	-	-
222	465	0.390	Undecane	1120-21-4	$C_{11}H_{24}$	1	1106	1101	-	[23]
223	465	0.650	1,9-Dodecadiene	-	$C_{12}H_{22}$	2	1106	-	-	-
224	520	0.890	7-Methyl-2-decene	74630-23-2	$C_{11}H_{22}$	2	1168	-	-	-
225	540	0.910	C12 (*m/z* 41, 55, 69, 43)	-	$C_{14}H_{28}$	3	1190	-	-	-
226	550	0.400	Dodecane	112-40-3	$C_{12}H_{26}$	1	1201	1201	-	[23]
227	635	0.410	Tridecane	629-50-5	$C_{13}H_{28}$	1	1301	1301	-	[15]
228	715	0.420	Tetradecane	629-59-4	$C_{14}H_{30}$	1	1401	1400	-	[39]
229	765	0.420	C14 (*m/z* 43, 57, 41, 71)	1560-95-8	$C_{15}H_{32}$	3	1463	-	-	-
230	790	0.470	1-Pentadecene	13360-61-7	$C_{15}H_{30}$	2	1494	-	1492	[66]
231	795	0.450	Pentadecane	629-62-9	$C_{15}H_{32}$	1	1501	1500	-	[39]
**232**	**880**	**0.490**	**Hexadecane**	**544-76-3**	**$C_{16}H_{34}$**	**1**	**1601**	**1600**	**-**	[39]
**233**	**965**	**0.430**	**Heptadecane**	**629-78-7**	**$C_{17}H_{36}$**	**1**	**1701**	**1700**	**-**	[39]
234	1010	0.440	Octadecane	593-45-3	$C_{18}H_{38}$	1	1754	1800	-	[39]
235	1150	0.450	Nonadecane	629-92-5	$C_{19}H_{40}$	1	1919	1900	-	[39]
			Aromatic							
**236**	**115**	**0.460**	**Benzene**	**71-43-2**	**C_6H_6**	**1**	**643**	**-**	**648**	[67]
**237**	**170**	**0.540**	**Toluene**	**108-88-3**	**C_7H_8**	**1**	**759**	**771**		[9]
238	240	0.580	Ethylbenzene	100-41-4	C_8H_{10}	2	860	-	866	[13]
**239**	**250**	**0.590**	**1,3-Dimethylbenzene**	**108-38-3**	**C_8H_{10}**	**2**	**871**	**-**	**874**	[13]
**240**	**270**	**0.640**	**1,2-Dimethylbenzene**	**95-47-6**	**C_8H_{10}**	**2**	**901**	**900**	**908**	[10]
241	300	0.570	1-Methylethylbenzene	98-82-8	C_9H_{12}	2	927	930	-	[9]
242	320	0.690	2-Propenylbenzene	300-57-2	C_9H_{10}	2	948	952	-	[9]
**243**	**325**	**0.580**	**Propylbenzene**	**103-65-1**	**C_9H_{12}**	**2**	**953**	**959**	**-**	[9]
244	330	0.470	*m/z* 91, 106, 77, 119	-	-	3	958	-	-	-
**245**	**335**	**0.590**	**1-Ethyl-4-methylbenzene**	**622-96-8**	**C_9H_{12}**	**2**	**964**	**970**	**-**	[9]
246	350	0.630	1-Ethyl-2-methylbenzene	611-14-3	C_9H_{12}	2	980	988	-	[9]
247	355	0.750	(1-Methylethenyl)benzene	98-83-9	C_9H_{10}	2	985	988	-	[9]
**248**	**365**	**0.640**	**1,3,5-Trimethylbenzene**	**108-67-8**	**C_9H_{12}**	**2**	**995**	**974**	**-**	[9]
249	370	0.770	1-Propenylbenzene	637-50-3	C_9H_{10}	2	1001	-	1000	[68]
**250**	**390**	**0.580**	**1-Methyl-2-(1-methylethyl)benzene**	**527-84-4**	**$C_{10}H_{14}$**	**2**	**1023**	**-**	**1022**	[69]
**251**	**390**	**0.690**	**1,2,3-Trimethylbenzene**	**526-73-8**	**C_9H_{12}**	**1**	**1023**	**-**	**1022**	[69]
252	420	0.590	1-Methyl-3-propylbenzene	1074-43-7	$C_{10}H_{14}$	2	1056	1058		[9]
**253**	**425**	**0.610**	**2-Ethyl-1,4-dimethylbenzene**	**1758-88-9**	**$C_{10}H_{14}$**	**2**	**1062**	**-**	**1087**	[70]
254	440	0.630	1-Ethyl-2,3-dimethylbenzene	933-98-2	$C_{10}H_{14}$	2	1079	1094	-	[9]
255	450	0.740	o-Isopropenyltoluene	7399-49-7	$C_{10}H_{12}$	2	1095	-	-	-
256	470	0.660	2-Ethyl-1,3-dimethylbenzene	1758-88-9	$C_{10}H_{14}$	2	1112	-	1087	[70]
257	505	0.750	1-Phenyl-1-butene	1005-64-7	$C_{10}H_{12}$	2	1151	-	-	-
258	480	0.670	1,2,4,5-Tetramethylbenzene	95-93-2	$C_{10}H_{14}$	2	1123	1130	-	[9]

259	485	0.970	Diethenylbenzene	1321-74-0	$C_{10}H_{10}$	2	1129	-	-	-
260	550	0.650	2,4-Diethyl-1-methylbenzene	1758-85-6	$C_{11}H_{16}$	2	1201	-	-	-
261	595	0.500	*m/z* 57, 175, 41, 91			3	1254	-	-	-
262	620	0.740	Pentamethylbenzene	700-12-9	$C_{11}H_{16}$	2	1283	-	1290	[71]
**263**	**700**	**1.270**	**Biphenyl**	**92-52-4**	$\mathbf{C_{12}H_{10}}$	**2**	**1383**	**-**	**1385**	[17]
264	815	1.260	Bibenzyl	103-29-7	$C_{14}H_{14}$	2	1525	-	1519	[34]
**265**	**880**	**1.020**	**2-Methyl-6-phenyl-1,6-heptadiene**	**51708-97-5**	$\mathbf{C_{14}H_{18}}$	**2**	**1601**	**-**	**-**	-
266	910	0.590	1,1-Diethylpropylbenzene	4170-84-7	$C_{13}H_{20}$	2	1636	-	-	-
267	920	0.590	1-Propylnonylbenzene	2719-64-4	$C_{18}H_{30}$	2	1648	-	-	-
268	925	1.270	1,1'-(1,3-Propanediyl)bis-benzene	1081-75-0	$C_{15}H_{16}$	2	1654	-	1633	[47]
269	990	0.590	1-Propylheptylbenzene	4537-12-6	$C_{16}H_{26}$	2	1730	-	-	-
270	1005	0.610	1-Pentylhexylbenzene	4537-14-8	$C_{17}H_{28}$	2	1748	-	-	-
271	1140	0.770	1-Methylnonylbenzene	4537-13-7	$C_{16}H_{26}$	2	1907	-	-	-
			Cyclic							
272	100	0.340	Methylcyclopentane	96-37-7	C_6H_{12}	2	611	627	-	[9]
273	215	0.730	Dicyclopropylmethane	5685-47-2	C_7H_{12}	2	830	-	-	-
274	235	0.430	1-Ethylcyclohexene	1453-24-3	C_8H_{14}	2	853	-	-	-
275	270	0.530	1,2-Dimethyl-1,4-cyclohexadiene	17351-28-9	C_8H_{12}	2	895	-	-	-
276	270	0.790	1,3,5,7-Cyclooctatetraene	629-20-9	C_8H_8	2	895	-	894	[72]
277	305	0.490	1,2-Propadienylcyclohexane	5664-17-5	C_9H_{14}	2	932	-	-	-
278	350	0.540	1-Ethyl-1,4-cyclohexadiene	19841-74-8	C_8H_{12}	2	980	-	-	-
279	495	1.030	1-Cyclohexylheptene	114614-83-4	$C_{13}H_{24}$	2	1140	-	-	-
280	515	0.810	1-Ethyl-2-methylcyclohexane	4923-78-8	C_9H_{18}	2	1162	-	-	-
281	695	0.670	(2-Ethyl-1-methyl-1-butenyl)cyclohexane	74810-42-7	$C_{13}H_{24}$	2	1376	-	-	-
282	755	0.460	Isobutylcyclopentane	3788-32-7	C_9H_{18}	2	1451	-	-	-
283	840	0.480	Nonylcyclohexane	2883-02-5	$C_{15}H_{30}$	2	1554	-	1556	[73]
			**Ketones**							
			Aliphatic							
**284**	**75**	**0.390**	**2-Propanone**	**67-64-1**	$\mathbf{C_3H_6O}$	**1**	**559**	**-**	**503**	[8]
285	90	0.440	2-Butanone	78-93-3	C_4H_8O	1	590	601	-	[26]
286	90	0.480	3-Buten-2-one	78-94-4	C_4H_6O	2	590	-	581	[74]
287	90	0.560	2,3-Butanedione	431-03-8	$C_4H_6O_2$	2	591	-	592	[75]
288	125	0.500	2-Pentanone	107-87-9	$C_5H_{10}O$	2	664	-	686	[76]
289	125	0.650	2,3-Pentanedione	600-14-6	$C_5H_8O_2$	2	665	-	693	[13]
290	130	0.500	3-Pentanone	96-22-0	$C_5H_{10}O$	2	675	-	700	[7]
291	140	1.540	3-Hydroxy-2-butanone	513-86-0	$C_4H_8O_2$	2	698	733	-	[15]
292	150	0.770	3-Penten-2-one	3102-33-8	C_5H_8O	2	717	-	735	[7]
293	160	0.510	3-Methyl-2-pentanone	565-61-7	$C_6H_{12}O$	2	738	750	-	[9]
294	190	0.690	4-Methyl-3-penten-2-one	141-79-7	$C_6H_{10}O$	2	801	801	-	[10]
295	240	0.610	5-Methyl-2-hexanone	110-12-3	$C_7H_{14}O$	2	860	-	857	[77]
296	250	0.570	4-Heptanone	123-19-3	$C_7H_{14}O$	2	871	-	-	-

**297**	**265**	**0.580**	**3-Heptanone**	**106-35-4**	$\mathbf{C_7H_{14}O}$	**1**	**889**	**884**	**-**	[9]
298	270	0.620	2-Heptanone	110-43-0	$C_7H_{14}O$	2	895	895	-	[15]
299	275	0.740	5-Hepten-2-one	6714-00-7	$C_7H_{12}O$	2	901	-	866	[78]
300	350	0.690	1-Octen-3-one	4312-99-6	$C_8H_{14}O$	2	980	977	-	[9]
301	355	0.710	2,3-Octanedione	585-25-1	$C_8H_{14}O_2$	2	985	-	980	[13]
**302**	**355**	**0.740**	**6-Methyl-5-hepten-2-one**	**110-93-0**	$\mathbf{C_8H_{14}O}$	**1**	**985**	**985**	**-**	[9]
303	360	0.600	3-Octanone	106-68-3	$C_8H_{16}O$	1	990	-	989	[24]
304	360	0.640	2-Octanone	111-13-7	$C_8H_{16}O$	2	990	-	992	[7]
305	405	0.780	3-Octen-2-one	1669-44-9	$C_8H_{14}O$	2	1040	-	1046	[79]
306	415	0.640	5-Ethyl-2-heptanone	-	$C_9H_{18}O$	2	1051	-	-	-
307	435	0.680	3-Nonen-2-one (isomer)	14309-57-0	$C_9H_{16}O$	2	1073	-	1079	[55]
308	450	0.610	3-Nonanone	925-78-0	$C_9H_{18}O$	2	1090	-	1091	[80]
309	455	0.640	2-Nonanone	821-55-6	$C_9H_{18}O$	2	1095	1093	-	[26]
310	460	0.730	5-Nonen-2-one	27039-84-5	$C_9H_{16}O$	2	1101	-	-	-
311	470	1.040	6-Methyl-3,5-heptadiene-2-one	1604-28-0	$C_8H_{12}O$	2	1112	-	1110	[81]
312	490	0.630	*m/z* 43, 58, 71, 41	-	-	3	1134	-	-	-
**313**	**495**	**0.760**	**3-Nonen-2-one (isomer)**	**14309-57-0**	$\mathbf{C_9H_{16}O}$	**2**	**1140**	**-**	**1144**	[82]
314	530	0.690	1-Decen-3-one	-	$C_{10}H_{18}O$	2	1179	-	-	-
315	540	0.600	3-Decanone	928-80-3	$C_{10}H_{20}O$	2	1190	-	1186	[5]
316	545	0.640	2-Decanone	693-54-9	$C_{10}H_{20}O$	2	1195	1197	-	[23]
317	570	1.760	3,8-Nonadien-2-one	55282-90-1	$C_9H_{14}O$	2	1226	-	-	-
318	585	0.750	3-Decen-2-one	10519-33-2	$C_{10}H_{18}O$	2	1242	-	-	-
319	610	0.580	*m/z* 43, 58, 71, 72	-	-	3	1271	-	-	-
320	610	0.750	4,8-Dimethyl-nona-3,8-dien-2-one	-	$C_{11}H_{18}O$	2	1272	-	-	-
321	620	0.680	3-Undecen-2-one	-	$C_{11}H_{20}O$	2	1283	-	-	-
322	625	0.620	3-Undecanone	2216-87-7	$C_{11}H_{22}O$	2	1289	-	1283	[83]
323	625	0.720	5-Ethyl-4-methyl-5-hepten-3-one	74764-56-0	$C_{10}H_{18}O$	2	1289	-	-	-
324	630	0.640	2-Undecanone	112-12-9	$C_{11}H_{22}O$	2	1295	1291	-	[23]
325	650	0.560	5-Dodecanone	19780-10-0	$C_{12}H_{24}O$	2	1319	-	-	-
326	670	0.630	*m/z* 43, 58, 57, 71	-	-	3	1345	-	-	-
327	690	0.600	2-Methyl-5-undecanone	50639-02-6	$C_{12}H_{24}O$	2	1370	-	-	-
328	695	0.820	Tridecane-2,4-dione	25276-80-6	$C_{13}H_{24}O_2$	2	1376	-	-	-
329	710	0.680	2-Dodecanone	6175-49-1	$C_{12}H_{24}O$	2	1395	1398	-	[23]
330	735	0.590	6-Tridecanone	22026-12-6	$C_{13}H_{26}O$	2	1426	-	-	-
**331**	**755**	**0.800**	**6,10-Dimethyl-,5,9-undecadien-2-one**	**3796-70-1**	$\mathbf{C_{13}H_{22}O}$	**2**	**1451**	**-**	**1455**	[48]
332	770	0.630	*m/z* 58, 43, 71, 57	-	-	3	1470	-	-	-
333	790	0.690	2-Tridecanone	593-08-8	$C_{13}H_{26}O$	2	1495	1498	-	[23]
			Aromatic							
334	435	1.500	Acetophenone	98-86-2	C_8H_8O	1	1074	1093	-	[23]
335	490	1.460	1-Phenyl-2-propanone	103-79-7	$C_9H_{10}O$	2	1135	-	1124	[84]
336	530	1.320	1-(4-Methylphenyl)ethanone	122-00-9	$C_9H_{10}O$	2	1179	-	1182	[5]
337	595	1.140	1-Phenyl-1-butanone	495-40-9	$C_{10}H_{12}O$	2	1254	-	-	-

338	650	1.070	4-Isopropylacetophenone	645-13-6	$C_{11}H_{14}O$	2	1320	-	-	-
339	910	2.210	Benzophenone	119-61-9	$C_{13}H_{10}O$	1	1638	-	1621	[7]
			Cyclic							
340	270	0.890	Cyclohexanone	108-94-1	$C_6H_{10}O$	1	895	-	895	[7]
341	295	3.000	Butyrolactone	96-48-0	$C_4H_6O_2$	1	924	-	920	[25]
342	305	0.740	1-Cyclopentylethanone	6004-60-0	$C_7H_{12}O$	2	932	933	-	[9]
343	310	0.760	2-Ethylcyclopentanone	4971-18-0	$C_7H_{12}O$	2	938	-	-	-
344	405	0.750	Cyclooctanone	502-49-8	$C_8H_{14}O$	2	1040	-	-	-
345	495	0.740	2-Hexylcyclopentanone	13074-65-2	$C_{11}H_{20}O$	2	1140	-	-	-
346	515	0.790	3-Butylcyclopentanone	57283-81-5	$C_9H_{16}O$	2	1162	-	-	-
347	525	0.840	2-Ethylcycloheptanone	3183-41-3	$C_9H_{16}O$	2	1173	-	-	-
348	565	0.870	Cyclononanone	3350-30-9	$C_9H_{16}O$	2	1219	-	1239	[85]
349	605	0.780	3,3,5-Trimethylcyclohexanone	873-94-9	$C_9H_{16}O$	2	1266	-	1285	[86]
			***N*-compounds**							
350	145	0.770	2-Nitropropane	79-46-9	$C_3H_7NO_2$	2	707	-	-	-
351	170	0.880	Pyridine	110-86-1	C_5H_5N	1	760	-	753	[7]
352	195	0.970	2-Methylpyridine	109-06-8	C_6H_7N	2	807	-	821	[76]
353	360	1.860	Benzonitrile	100-47-0	C_7H_5N	2	991	988	-	[9]
354	405	0.500	Cyclobutylamine	2516-34-9	C_4H_9N	2	1039	-	-	-
355	405	1.220	6-Nitro-2-hexene	40244-96-0	$C_6H_{11}NO_2$	2	1040	-	-	-
356	415	0.990	1-Nitrohexane	646-14-0	$C_6H_{13}NO_2$	2	1051	-	1050	[77]
357	445	0.710	3-Nitro-1-butene	-	$C_4H_7NO_2$	2	1084	-	-	-
358	490	1.470	*N*-Ethyl-benzenamine	103-69-5	$C_8H_{11}N$	2	1135	-	-	-
359	640	0.900	*N*,*N*-Dibutyl-formamide	761-65-9	$C_9H_{19}NO$	2	1307	-	1319	[87]
360	655	0.700	2,5-Dimethyl-4-nitro-3-hexanone	59906-54-6	$C_8H_{15}NO_3$	2	1326	-	-	-
361	955	0.810	2,4-di-*t*-Butyl-6-nitro-phenol	-	$C_{14}H_{21}NO_3$	2	1689	-	-	-
362	1015	1.120	3-(4-Nitrophenyl)pentan-3-ol	-	$C_{11}H_{15}NO_3$	2	1760	-	-	-
			***S*-compounds**							
363	150	1.260	Thiazole	288-47-1	C_3H_3NS	1	718	-	735	[76]
364	155	0.620	Dimethyldisulfide	624-92-0	$C_2H_6S_2$	1	728	748	-	[9]
365	170	0.640	2-Methylthiophene	554-14-3	C_5H_6S	2	759	-	775	[76]
366	200	0.960	2-Methylthiazole	3581-87-1	C_4H_5NS	2	813	-	808	[76]
367	285	1.540	3-(Methylthio)propanal	3268-49-3	C_4H_8OS	2	912	-	904	[13]
368	340	0.920	Dimethyltrisulfide	3658-80-8	$C_2H_6S_3$	2	969	-	969	[88]
369	390	1.850	2-Acetylthiazole	24295-03-2	C_5H_5NOS	2	1024	-	1014	[5]
370	425	0.650	1-(3-Thienyl)-2-propanone	-	C_7H_8OS	2	1062	-	-	-
371	445	1.310	Benzenemethanethiol	100-53-8	C_7H_8S	2	1085	-	1080	[89]
372	480	1.530	2-Propionylthiazole	-	C_6H_7NOS	2	1124	-	-	-
373	520	1.080	(Methylthio)methylbenzene	766-92-7	$C_8H_{10}S$	2	1168	-	1167	[77]
374	545	1.440	Benzo[b]thiophene	95-15-8	C_8H_6S	2	1196	-	1172	[90]
375	575	1.960	Benzothiazole	95-16-9	C_7H_5NS	2	1232	1223	-	[26]

376	580	0.880	Cyclohexyl isothiocyanate	1122-82-3	$C_7H_{11}NS$	2	1236	-	-	-
377	590	0.660	2-Tertiobutylthiophene	-	$C_8H_{12}S$	2	1248	-	-	-
378	675	0.420	Cyclohexylmethylbutyl sulfite	-	$C_{11}H_{22}O_3S$	2	1351	-	-	-
379	680	0.540	S-Methyl-oct-2-enethioate	91944-66-0	$C_9H_{16}OS$	2	1357	-	-	-
380	950	0.460	Cyclohexylmethyl hexyl sulfite	-	$C_{13}H_{26}O_3S$	2	1683	-	-	-
			**Terpenic compounds**							
			Monoterpenenic compounds							
			Hydrocarbon-type							
381	310	0.410	α-Pinene	7785-26-4	$C_{10}H_{16}$	1	937	959	-	[91]
382	330	0.480	Verbenene	4080-46-0	$C_{10}H_{14}$	2	958	963	-	[92]
383	375	0.480	α-Phellandrene	99-83-2	$C_{10}H_{16}$	1	1006	1007	-	[92]
384	395	0.500	Limonene	138-86-3	$C_{10}H_{16}$	1	1028	1027	-	[92]
385	450	0.610	3-Methyl-4-methylenebicyclo[3.2.1]oct-2-ene	49826-53-1	$C_{10}H_{14}$	2	1090	-	-	-
386	485	0.790	2,6-Dimethylbicyclo[3.2.1]octane	-	$C_{10}H_{18}$	2	1129	-	-	-
387	695	0.680	6,6-dimethyl-3-methylenebicyclo[3.1.1]heptane	16022-04-1	$C_{10}H_{16}$	2	1376	-	-	-
			Oxygen-containing compounds							
388	400	0.500	1,8-Cineole	470-82-6	$C_{10}H_{18}O$	1	1034	1041	-	[92]
**389**	**435**	**0.790**	**2,6-Dimethyl-7-octen-2-ol**	**18479-58-8**	$\mathbf{C_{10}H_{20}O}$	**2**	**1073**	**-**	**1075**	[93]
390	445	0.700	2,6-Dimethyl-1,7-octadien-3-ol	22460-59-9	$C_{10}H_{18}O$	2	1084	1095	-	[91]
391	460	0.670	Dihydrolinalool	78-69-3	$C_{10}H_{22}O$	2	1101	-	1097	[7]
392	460	0.700	2,6-Dimethyl-2-octanol	18479-57-7	$C_{10}H_{22}O$	2	1101	-	-	-
393	465	0.910	Linalool	78-70-6	$C_{10}H_{18}O$	1	1107	1107	-	[92]
394	480	0.940	Fenchyl alcohol	1632-73-1	$C_{10}H_{18}O$	2	1123	1121	-	[92]
395	500	1.090	Pinocarveol	547-61-5	$C_{10}H_{16}O$	2	1146	1148	-	[92]
396	505	0.780	Camphor	464-48-2	$C_{10}H_{16}O$	1	1151	1147	-	[94]
397	505	1.150	Verbenol	473-67-6	$C_{10}H_{16}O$	2	1151	-	1147	[95]
398	510	0.670	*p*-Menthan-3-one	491-07-6	$C_{10}H_{18}O$	2	1156	1158	-	[32]
399	520	0.840	Pinocarvone	30460-92-5	$C_{10}H_{14}O$	2	1168	1168	-	[92]
400	525	1.120	Borneol	507-70-0	$C_{10}H_{18}O$	1	1174	1172	-	[96]
401	530	0.920	Menthol	2216-51-5	$C_{10}H_{20}O$	1	1179	-	1172	[97]
402	540	1.660	*p*-Cymen-8-ol	1197-01-9	$C_{10}H_{14}O$	2	1191	1203	-	[96]
403	545	0.910	Dihydrocitronellol	106-21-8	$C_{10}H_{22}O$	2	1196	-	1196	[98]
404	545	1.020	α-Terpineol	98-55-5	$C_{10}H_{18}O$	1	1196	1201	-	[92]
405	550	0.870	Myrtenal	564-94-3	$C_{10}H_{14}O$	2	1201	1204	-	[96]
406	560	1.040	Verbenone	1196-01-6	$C_{10}H_{14}O$	1	1213	-	1204	[54]
407	595	0.620	Linalyl acetate	115-95-7	$C_{12}H_{20}O_2$	2	1254	-	1257	[54]
**408**	**625**	**0.630**	**Endobornyl acetate**	**76-49-3**	$\mathbf{C_{12}H_{20}O_2}$	**2**	**1289**	**-**	**1285**	[5]
409	675	0.680	β-Terpenyl acetate	10198-23-9	$C_{12}H_{20}O_2$	2	1351	-	-	-
410	700	0.730	Geraniol acetate	105-87-3	$C_{12}H_{20}O_2$	1	1382	-	1383	[99]
411	735	0.910	Verdyl acetate	5413-60-5	$C_{12}H_{16}O_2$	2	1426	-	-	-

			**Sesquiterpenes**							
			Hydrocarbon-type							
412	725	0.530	Valencene	4630-07-3	$C_{15}H_{24}$	1	1413	-	-	-
413	725	0.540	Longifolene	475-20-7	$C_{15}H_{24}$	2	1413	1395	-	[96]
414	820	0.600	δ-Cadinene	483-76-1	$C_{15}H_{24}$	2	1530	1511	-	[96]
415	820	0.690	Calamenene	483-77-2	$C_{15}H_{22}$	2	1530	1525	-	[100]
416	820	0.830	α-Bisabolene	29837-07-8	$C_{15}H_{24}$	2	1530	1537	-	[92]
417	835	0.770	α-Calacorene	21391-99-1	$C_{15}H_{20}$	2	1548	1542	-	[100]
418	840	0.870	Patchulane	19078-35-4	$C_{15}H_{26}$	2	1554	-	-	-
419	1100	0.670	4,5,9,10-Dehydro-isolongifolene	-	$C_{15}H_{20}$	2	1860	-	-	-
			Oxygen-containing compounds							
420	850	0.920	Nerolidol	7212-44-4	$C_{15}H_{26}O$	1	1566	1568	-	[96]
421	855	0.960	Longicamphenylone	-	$C_{14}H_{22}O$	2	1572	-	-	-
422	880	0.780	Torreyol	19435-97-3	$C_{15}H_{26}O$	2	1601	-	1618	[101]
423	890	1.000	Cedrol	77-53-2	$C_{15}H_{26}O$	2	1613	1600	-	[92]
424	920	1.020	τ-Cadinol	5937-11-1	$C_{15}H_{26}O$	2	1648	1637	-	[100]
425	1175	0.820	α-Bisabolene epoxide	-	$C_{15}H_{24}O$	2	1951	-	-	-
			**Norisoprenoids**							
426	575	1.080	Tetrahydroionol	4361-23-3	$C_{13}H_{26}O$	2	1231	-	-	-
427	645	0.610	Edulan	41678-30-2	$C_{13}H_{20}O$	2	1313	-	-	-
**428**	**780**	**0.750**	**α-Methylionone**	**127-51-5**	$\mathbf{C_{14}H_{22}O}$	**2**	**1482**	**-**	**1481**	[102]

References

1. Sumner, L. et al. Proposed minimum reporting standards for Chemical analysis. *Metabolomics*. **3**, 211–221 (2007).
2. Kotseridis, Y. & Baumes, R. Identification of impact odorants in Bordeaux red grape juice, in the commercial yeast used for its fermentation, and in the produced wine. *J Agric Food Chem*. **48**, 400-406 (2000).
3. Schnermann, P. & Schieberle, P. Evaluation of Key Odorants in Milk Chocolate and Cocoa Mass by Aroma Extract Dilution Analyses. *J Agric Food Chem*. **45**, 867-872 (1997).
4. Figuérédo, G., Cabassu, P., Chalchat, J.C. & Pasquier, B. Studies of Mediterranean oregano populations. VIII—Chemical composition of essential oils of oreganos of various origins. *Flavour Fragr J*. **21**, 134-139 (2006).
5. Adams, R.P. Identification of essential oil components by gas chromatography/mass spectrometry. Carol Stream, IL. Allured Publishing Corporation, (1995).
6. Wu, S., Zorn, H., Krings, U. & Berger, R.G. Volatiles from submerged and surface-cultured beefsteak fungus, *Fistulina hepatica*. *Flavour Fragr J.* **22**, 53-60 (2007).
7. Pino, J.A., Mesa, J., Munoz, Y., Marti, M.P. & Marbot, R. Volatile components from mango (*Mangifera indica* L.) cultivars. *J Agric Food Chem*. **53**, 2213-2223 (2005).
8. Rembold, H., Wallner, P., Nitz, S., Kollmannsberger, H. & Drawert, F. Volatile components of chickpea (Cicer arietinum L.) seed. *J Agric Food Chem*. **37**, 659-662 (1989).
9. Xu, X. et al. Comprehensive two-dimensional gas chromatography (GC × GC) measurements of volatile organic compounds in the atmosphere. *Atmos Chem Phys*. **3**, 665-682 (2003).
10. Rocha, S.M. et al. Exploring the potentialities of comprehensive two-dimensional gas chromatography coupled to time of flight mass spectrometry to distinguish bivalve species: Comparison of two clam species (*Venerupis decussata* and *Venerupis philippinarum*). *J Chromatogr A*. **1315**, 152–161 (2013).
11. Guichard, E. & Souty, M. Comparison of the relative quantities of aroma compounds found in fresh apricot (*Prunus armeniaca*) from six different varieties. *Z Lebensm Unters Forsch.* **186**, 301-307 (1988).

12. Rodriguez-Burruezo, A., Kollmannsberger, H., Prohens, J., Nitz, S. & Nuez, F. Analysis of the volatile aroma constituents of parental and hybrid clones of pepino (*Solanum muricatum*). *J Agric Food Chem*. **52**, 5663-5669 (2004).
13. Engel, E. & Ratel, J. Correction of the data generated by mass spectrometry analyses of biological tissues: application to food authentication. *J Chromatogr A*. **1154**, 331-341 (2007).
14. Turchimi, G.N. et al. Effects of dietary lipid sources on flavour volatile compounds of brown trout (*Salmo trurra* L.) fillet. *J Appl Ichthyol*. **20**, 71-75 (2004).
15. Salvador, Â.C. et al. Can Volatile Organic Metabolites Be Used to Simultaneously Assess Microbial and Mite Contamination Level in Cereal Grains and Coffee Beans? *PLoS ONE*. **8**, e59338 (2013).
16. Boylston, T.D. & Viniyard, B.T. Isolation of volatile flavor compounds from peanut butter using purge-and-trap technique in Instrumental Methods in Food and Beverage Analysis. D. Wetzel and G. Charalambous, ed(s), 225-243 (1998).
17. Leffingwell, J.C. & Alford, E. Volatile constituents of perique tobacco. *Electron J Environ Agric Food Chem*. **4**, 899-915 (2005).
18. Zhao, Y. et al. Extraction, preparation and identification of volatile compounds in Changyu XO brandy. *Se Pu*. **26**, 212-222 (2008).
19. Berdague, J.L., Tournayre, P. & Cambou, S. Novel multi-gas chromatography-olfactometry device and software for the identification of odour-active compounds. *J Chromatogr A*. **1146**, 85-92 (2007).
20. Mahmood, U, Kaul, VK & Acharya, R. Volatile constituents of *Capillipedium parviflorum*. *Phytochemistry*. **65**, 2163-2166 (2004).
21. Forero, M.D., Quijano, C.E. & Pino, J.A. Volatile compounds of chile pepper (*Capsicum annuum* L. var. *glabriusculum*) at two ripening stages. *Flavour Fragr J*. **24**, 25-30 (2009).
22. Nogueira, P.C., Bittrich, V., Shepherd, G.J., Lopes, A.V. & Marsaioli, A.J. The ecological and taxonomic importance of flower volatiles of *Clusia* species (Guttiferae). *Phytochemistry*. **56**, 443-452 (2001).
23. Silva, I, Rocha, S.M., Coimbra, M.A. & Marriott, P.J. Headspace solid-phase microextraction combined with comprehensive two-dimensional gas chromatography time-of-flight mass spectrometry for the determination of volatile compounds from marine salt. *J Chromatogr A*. **1217**, 5511-21 (2010).
24. Zhao, C. et al. Comparative analysis of chemical components of essential oils from different samples of Rhododendron with the help of chemometrics methods. *Chemometr Intell Lab Syst*. **82**, 218-228 (2006).
25. Pino, J.A., Marbot, R., Rosado, A. & Vázquez, C. Volatile constituents of Malay rose apple [*Syzygium malaccense* (L.) Merr. & Perry]. *Flavour Fragr J*. **19**,32-35 (2004).
26. Caldeira, M. et al. Profiling allergic asthma volatile metabolic patterns using a headspace-solid phase microextraction/gas chromatography based methodology. *J Chromatogr A*. **1218**, 3771-3780 (2011).
27. Dallüge, J. et al. Unravelling the composition of very complex samples by comprehensive gas chromatography coupled to time-of-flight mass spectrometry: Cigarette smoke. *J Chromatogr A*. **974**, 169-184 (2002).
28. Eyres, G., Dufour, J.P., Hallifax, G., Sotheeswaran, S. & Marriott, P.J. Identification of character-impact odorants in coriander and wild coriander leaves using gas chromatography-olfactometry (GCO) and comprehensive two-dimensional gas chromatography-time-of-flight mass spectrometry (GC x GC-TOFMS). *J Sep Sci*. **28**, 1061-1074 (2005).
29. Setzer, W.N., Noletto, J.A., Lawton, R.O. & Haber, W.A. Leaf essential oil composition of five Zanthoxylum species from Monteverde, Costa Rica. *Mol Divers*. **9**, 3-13 (2005).
30. Dickschat, J.S., Bode, H.B., Wenzel, S.C., Muller, R. & Schulz, S. Biosynthesis and identification of volatiles released by the myxobacterium *Stigmatella aurantiaca*. *ChemBioChem*. **6**, 2023-2033 (2005).
31. Robinson, A.L, Boss, P.K., Heymann, H., Solomon, P.S. & Trengove, R.D. Development of a sensitive non-targeted method for characterizing the wine volatile profile using headspace solid-phase microextraction comprehensive two-dimensional gas chromatography time-of-flight mass spectrometry. *J Chromatogr A*. **1218**, 504-517 (2001).
32. Kallio, M. et al. Comprehensive two-dimensional gas chromatography coupled to time-of-flight mass spectrometry in the identification of organic compounds in atmospheric aerosols from coniferous forest. *J Chromatogr A*. **1125**, 234-243 (2006).
33. Weldegergis, B.T., Crouch, A.M., Gorecki, T. & de Villiers, A. Solid phase extraction in combination with comprehensive two-dimensional gas chromatography coupled to time-of-flight mass spectrometry for the detailed investigation of volatiles in South African red wines. *Anal Chim Acta*. **701**, 98-111 (2001).
34. Song, C., Lai, W.-C., Madhusudan, R., Boli Wei, K. & Wei, B. Temperature-Programmed Retention Indices for GC and GC-MS of Hydrocarbon Fuels and Simulated Distillation GC of Heavy Oils. In: Hsu, C. (ed) Analytical Advances for Hydrocarbon Research SE - 7. Springer US; 147-210 (2003).

35. Aaslyng, M.D., Elmore, J.S. & Mottram, D.S. Comparison of the aroma characteristics of acid-hydrolyzed and enzyme-hydrolyzed vegetable proteins produced from soy. *J Agric Food Chem*. 1998; **46**, 5225-5231 (1998).
36. Qian, M. & Reineccius, G. Potent aroma compounds in Parmigiano Reggiano cheese studied using a dynamic headspace (purge-trap) method. *Flavour Fragr J*. **18**, 252-259 (2003).
37. Loureiro, C.C. et al. Urinary metabolomic changes as a predictive biomarker of asthma exacerbation. *J Allergy Clin Immunol*. **133**, 261-263 (2014).
38. Bruna, J.M. et al. The contribution of *Penicillium aurantiogriseum* to the volatile composition and sensory quality of dry fermented sausages. *Meat Sci*. **59**, 97-107 (2001).
39. Rocha, S.M. et al. Exploring the human urine metabolomic potentialities by comprehensive two-dimensional gas chromatography coupled to time of flight mass spectrometry. *J Chromatogr A*. **1252**, 155-163 (2012).
40. Ruther, J. Retention index database for identification of general green leaf volatiles in plants by coupled capillary gas chromatography−mass spectrometry. *J Chromatogr A*. **890**, 313-319 (2000).
41. Avato, P., Raffo, F., Aldouri, N.A. & Vartanian, S.T. Essential oils of Varthemia iphionoides from Jordan. *Flavour Fragr J*. **19**, 559-561 (2004).
42. Adams, R.P. Systematics of smooth leaf margin *Juniperus* of the western hemisphere based on leaf essential oils and RAPD DNA fingerprinting. *Biochem Syst Ecol*. **28**, 149-162 (2000).
43. Lazari, D.M, Skaltsa, H.D. & Constantinidis, T. Volatile constituents of *Cerastium candidissimum*, a Greek endemic species. *Flavour Fragr J*. **15**, 174-176 (2000).
44. Spadone, J.C., Takeoka, G. & Liardon, R. Analytical investigation of Rio off-flavor in green coffee. *J Agric Food Chem*. **38**, 226-233 (1990).
45. Ramarathnam, N., Rubin, L.J. & Diosady, L.L. Studies on meat flavor. 3. A novel method for trapping volatile components from uncured and cured pork. *J Agric Food Chem*. **41**, 933-938 (1993).
46. Lizárraga-Guerra, R., Guth, H. & López, M.G. Identification of the most potent odorants in huitlacoche (*Ustilago maydis*) and austern pilzen (*Pleurotus* sp.) by aroma extract dilution analysis and static head-space samples. *J Agric Food Chem*. **45**, 1329-1332 (1997).
47. Shapi, M.M. & Hesso, A. Thermal decomposition of polystyrene: Volatile compounds from large-scale pyrolysis. *J Anal Appl Pyrolysis*. **18**, 143-161 (1990).
48. Adams, R.P., Morris, J.A., Pandey, R.N. & Schwarzbach, A.E. Cryptic speciation between *Juniperus deltoides* and *Juniperus oxycedrus* (Cupressaceae) in the Mediterranean. *Biochem Syst Ecol*. **33**, 771-787 (2005).
49. Bieri, S. & Marriott, P.J. Dual-injection system with multiply injections for determining sidimentional retention indexes in comprehensive two-dimensional gas chromatography. *Anal Chem*. **80**,760-768 (2008).
50. Radulovic, N., Blagojevic, P. & Palic, R. Comparative study of the leaf volatiles of *Arctostaphylos uva-ursi* (L.) Spreng. and *Vaccinium vitis-idaea* L. (Ericaceae). *Molecules*. **15**, 6168-6185 (2010).
51. El-Sayed, A., Heppelthwaite, V., Manning, L., Gibb, A. & Suckling, D. Volatile constituents of fermented sugar baits and their attraction to lepidopteran species. *J Agric Food Chem*. **53**, 953-958 (2005).
52. de Souza, M.D., Vasquez, P., Del Mastro, N.L., Acree, T.E. & Lavin, E.H. Characterization of cachaca and rum aroma. *J Agric Food Chem*. **54**, 485-488 (2006).
53. Steinhaus, P. & Schieberle, P. Characterization of the key aroma compounds in soy sauce using approaches of molecular sensory science. *J Agric Food Chem*. **55**, 6262-6269 (2007).
54. Quijano, C.E., Salamanca, G. & Pino, J.A. Aroma volatile constituents of Colombian varieties of mango (*Mangifera indica* L.). *Flavour Fragr J*. **22**, 401-406 (2007).
55. Moio, L., Piombino, P. & Addeo, F. Odour-impact compounds of Gorgonzola cheese. *J Dairy Res*. **67**, 273-85 (2000).
56. Bauchot, A.D., Mottram, D.S., Dodson, A.T. & John, P. Effect of Aminocyclopropane-1-carboxylic Acid Oxidase Antisense Gene on the Formation of Volatile Esters in Cantaloupe Charentais Melon (Cv. Védrandais). *J Agric Food Chem*. **46**, 4787-4792 (1998).
57. Fernando, L.N. & Grün, I.U. Headspace–SPME analysis of volatiles of the ridge gourd (*Luffa acutangula*) and bitter gourd (*Momordica charantia*) flowers. *Flavour Frag J*. **16**,289-293 (2001).
58. Pino, J.A., Marbot, R. & Vazquez, C. Volatile components of tamarind (*Tamarindus indica* L.) grown in Cuba. *J Essent Oil Res*. **16**, 318-320 (2004).
59. Zellner, Bd'A. et al. Linear retention indices in gas chromatographic analysis: a review. *Flavour Frag J*. **23**, 297-314 (2008).
60. Wang, H., Guo, Y., Zhang, Z. & An, D. Fast analysis of volatile compounds in natural essences by automatic Static-Headspace-GC-MS. *J Instrum Anal*. **23**, 9-13 (2004).
61. Wu, S., Zorn, H., Krings, U. & Berger, R.G. Characteristic volatiles from young and aged fruiting bodies of wild Polyporus sulfureus (Bull.:Fr.) Fr. *J Agric Food Chem*. **53**, 4524-8 (2005).
62. Tzakou, O., Harvala, C., Galati, E.M. & Sanogo, R. Essential oil composition of Nepeta argolica Borey et Chaub. subsp. argolica. *Flavour Frag J*. **15**, 115-118 (2000).

63. Zaikin, V.G. Personal communication: Retention indices measured during 2010.
64. Zoghbi, M.D., Andrade, E.H. & Maia, J.G. Volatile constituents from leaves and flowers of *Alpinia speciosa* K. Schum. and *A. purpurata* (Viell.) Schum. *Flavour Frag J*. **14**, 411-414 (1999).
65. Larsen, T.O. & Frisvad, J.C. Characterization of volatile metabolites from 47 *Penicillium* taxa. *Mycol Res*. **99**, 1153-1166 (1995).
66. Flamini, G., Cioni, P.L. & Morelli, I. Composition of the essential oils and in vivo emission of volatiles of four *Lamium* species from Italy: *L. purpureum*, *L. hybridum*, *L. bifidum* and *L. amplexicaule*. *Food Chem*. **91**, 63-68 (2005).
67. Isidorov, V.A., Vinogorova, V.T. & Rafałowski, K. HS-SPME analysis of volatile organic compounds of coniferous needle litter. *Atmos Environ*. **37**, 4645-4650 (2003).
68. Buchin, S. et al. Identification de composés monoterpéniques, sesquiterpéniques et benzéniques dans un lait d'alpage très riche en ces substances. *Mitt aus Lebensmittelunters Hyg*. **93**, 199-216. (2002).
69. Adams, R.P. Systematics of the one seeded *Juniperus* of the eastern hemisphere based on leaf essential oils and random amplified polymorphic DNAs (RAPDs). *Biochem Syst Ecol*. 2000; **28**, 529-543 (2000).
70. Wang, Z., Fingas, M. & Li, K. Fractionation of a Light Crude Oil and Identification and Quantitation of Aliphatic, Aromatic, and Biomarker Compounds by GC-FID and GC-MS, Part II. *J Chromatogr Sci*. **32**, 367-382 (1994).
71. Wang, Z. & Fingas, M. Differentiation of the source of spilled oil and monitoring of the oil weathering process using gas chromatography-mass spectrometry. *J Chromatogr A*. **712**, 321-343 (1995).
72. Wang, H.Y. & Guo, Y.L. Rapid Analysis of the Volatile Compounds in the Rhizomes of *Rhodiola sachalinensis* and *Rhodiola sacra* by Static Headspace-Gas Chromatography–Tandem Mass Spectrometry. *Anal Lett*. **37**, 2151-2161 (2004).
73. Kenig, F. et al. Structure and distribution of branched aliphatic alkanes with quaternary carbon atoms in Cenomanian and Turonian black shales of Pasquia Hills (Saskatchewan, Canada). *Org Geochem*. **36**, 117-138 (2005).
74. Isidorov, V., Purzyńska, A., Modzelewska, A. & Serowiecka, M. Distribution coefficients of aliphatic alcohols, carbonyl compounds and esters between air and Carboxen/polydimethylsiloxane fiber coating. *Anal Chim Acta*. **560**,103-109 (2006).
75. Rychlik, M., Grosch, W. & Schieberle, P. Compilation of odor thresholds, odor qualities and retention indices of key food odorants. Garching, Deutsche Forschungsanstat für Lebensmittelchemie and Instit für Lebensmittelchemie der Technischen Universität München (1998).
76. Methven, L., Tsoukka, M., Oruna-Concha, M.J., Parker, J.K. & Mottram, D.S. Influence of Sulfur Amino Acids on the Volatile and Nonvolatile Components of Cooked Salmon (*Salmo salar*). *J Agric Food Chem*. **55**, 1427-1436 (2007).
77. Solina, M., Baumgartner, P., Johnson, R.L. & Whitfield, F.B. Volatile aroma components of soy protein isolate and acid-hydrolysed vegetable protein. *Food Chem*. **90**, 861-873 (2005).
78. Moio, L. & Addeo, F. Grana Padano cheese aroma. *J Dairy Res*. **65**, 317-333 (1998).
79. Qiming, X., Haidong, C., Huixian, Z. & Daqiang, Y. Chemical composition of essential oils of two submerged macrophytes, *Ceratophyllum demersum* L. and *Vallisneria spiralis* L. *Flavour Fragr J*. **21**, 524-526 (2006).
80. Dickschat, J.S., Wenzel, S.C., Bode, H.B., Müller, R. & Schulz, S. Biosynthesis of volatiles by the myxobacterium *Myxococcus xanthus*. *ChemBioChem*. **5**, 778-787 (2004).
81. Jalali-Heravi, M., Zekavat, B. & Sereshti, H. Characterization of essential oil components of Iranian geranium oil using gas chromatography–mass spectrometry combined with chemometric resolution techniques. *J Chromatogr A*. **1114**, 154-163 (2006).
82. Elmore, J.S., Campo, M.M., Enser, M. & Mottram, D.S. Effect of lipid composition on meat-like model systems containing cysteine, ribose, and polyunsaturated fatty acids. *J Agric Food Chem*. **50**, 1126-1132 (2002).
83. Smelcerovic, A., Spiteller, M., Ligon, A.P., Smelcerovic, Z. & Raabe, N. Essential oil composition of *Hypericum* L. species from Southeastern Serbia and their chemotaxonomy. *Biochem Syst Ecol*. **35**, 99-113 (2007).
84. Ferhat, M.A., Tigrine-Kordjani, N., Chemat, S., Meklati, B.Y. & Chemat, F. Rapid Extraction of Volatile Compounds Using a New Simultaneous Microwave Distillation: Solvent Extraction Device. *Chromatographia*. **65**, 217-222 (2007).
85. Dhanda, J.S., Pegg, R.B. & Shand, P.J. Saskatchewan specialty livestock value-added program - Saskatchewan agri-food innovation fund (AFIF) Project 98000016 (2003).

86. Ventanas, S., Estevez, M., Andres, A.I. & Ruiz, J. Analysis of volatile compounds of Iberian dry-cured loins with different intramuscular fat contents using SPME-DED. *Meat Sci*. **79**, 172-180 (2008).
87. Garcia-Esteban, M., Ansorena, D., Astiasaran, I., Martin, D. & Ruiz, J. Comparison of simultaneous distillation extraction (SDE) and solid-phase microextraction (SPME) for the analysis of volatile compounds in dry-cured ham. *J Sci Food Agric*. **84**, 1364-1370 (2004).
88. Zhou, Q., Wintersteen, C.L. & Cadwallader, K.R. Identification and quantification of aroma-active components that contribute to the distinct malty flavor of buckwheat honey. *J Agric Food Chem*. **50**, 2016-2021 (2002).
89. Tellez, M.R. et al. Composition of the essential oil of *Lepidium meyenii* (Walp.). *Phytochemistry*. **61**, 149-155 (2002).
90. Andersson, J.T. & Weis, U. Gas chromatographic determination of polycyclic aromatic compounds with fluorinated analogues as internal standards. *J Chromatogr A*. **659**, 151-161 (1994).
91. Rocha, S.M., Coelho, E., Zrostlikova, J., Delgadillo, I. & Coimbra, M.A. Comprehensive two-dimensional gas chromatography with time-of-flight mass spectrometry of monoterpenoids as a powerful tool for grape origin traceability. *J Chromatogr A*. **1161**, 292-299 (2007).
92. Petronilho S., Rocha, S.M., Ramírez-Chávez, E., Molina-Torres, J. & Rios-Chávez, P. Assessment of the terpenic profile of *Callistemon citrinus* (Curtis) Skeels from México. *Ind Crops Prod*. **46**, 369-379 (2013).
93. Diaz, A. & Kite, G. A comparison of the pollination ecology of *Arum maculatum* and *A. italicum* in England. *Watsonia*. **24**, 171-182 (2002).
94. Jalali, H.T. et al. Deeper insight into the monoterpenic composition of Ferula gummosa oleo-gum-resin from Iran. *Ind Crops Prod*. **36**, 500-507 (2012).
95. Lo Presti, M. et al. Evaluation of the volatile and chiral composition in *Pistacia lentiscus* L. essential oil. *Flavour Fragr J*. **23**, 249-257 (2008).
96. Jalali, H.T. et al. Assessment of the sesquiterpenic profile of *Ferula gummosa* oleo-gum-resin (galbanum) from Iran. Contributes to its valuation as a potential source of sesquiterpenic compounds. *Ind Crops Prod*. **44**, 185-191 (2013).
97. Sefidkon, F. & Jamzad, Z. Chemical composition of the essential oil of three Iranian *Satureja* species (*S. mutica*, *S. macrantha* and *S. intermedia*). *Food Chem*. **91**, 1-4 (2005).
98. Stashenko, E.E., Jaramillo, B.E. & Martínez, J.R. Comparación de la composición química y de la actividad antioxidante in vitro de los metabolitos secundarios volátiles de plantas de la familia Verbenaceae. *Rev Acad Colomb Cienc*. **27**, 579-597 (2003).
99. Adams, R.P. Systematics of multi-seeded eastern hemisphere *Juniperus* based on leaf essential oils and RAPD DNA fingerprinting. *Biochem Sys Ecol*. **27**, 709-725 (1999).
100. Petronilho, S., Maraschin, M., Delgadillo, I., Coimbra, M.A. & Rocha, S.M. Sesquiterpenic composition of the inflorescences of Brazilian chamomile (*Matricaria recutita* L.): Impact of the agricultural practices. *Ind Crops Prod*. **34**, 1482-1490 (2011).
101. Marongiu, B., Piras, A., Pani, F., Porcedda, S. & Ballero, M. Extraction, separation and isolation of essential oils from natural matrices by supercritical CO_2. *Flavour Fragr J*. **18**, 505-509 (2003).
102. Mondello, L. et al. Fast gas chromatography-full scan quadrupole mass spectrometry for the determination of allergens in fragrances. *J Sep Sci*. **30**, 1905-1911 (2007).
